# Advancing Precision: A Comprehensive Review of MRI Segmentation Datasets from BraTS Challenges (2012–2025)

**DOI:** 10.3390/s25061838

**Published:** 2025-03-15

**Authors:** Beatrice Bonato, Loris Nanni, Alessandra Bertoldo

**Affiliations:** Department of Information Engineering, University of Padova, Via Giovanni Gradenigo 6b, 35131 Padova, Italy; beatrice.bonato.4@studenti.unipd.it (B.B.); alessandra.bertoldo@unipd.it (A.B.)

**Keywords:** BraTS challenges, brain tumor segmentation, glioma, MRI imaging, dataset evolution, precision medicine, clinical applications

## Abstract

Brain Tumor Segmentation (BraTS) challenges have significantly advanced research in brain tumor segmentation and related medical imaging tasks. This paper provides a comprehensive review of the BraTS datasets from 2012 to 2024, examining their evolution, challenges, and contributions to MRI-based brain tumor segmentation. Over the years, the datasets have grown in size, complexity, and scope, incorporating refined pre-processing and annotation protocols. By synthesizing insights from over a decade of BraTS challenges, this review elucidates the progression of dataset curation, highlights the impact on state-of-the-art segmentation approaches, and identifies persisting limitations and future directions. Crucially, it provides researchers, clinicians, and industry stakeholders with a single, in-depth resource on the evolution and practical utility of BraTS datasets—demonstrating year-by-year improvements in the field and discussing their potential for enabling robust, clinically relevant segmentation methods that can further advance precision medicine. Additionally, an overview of the upcoming BraTS 2025 Challenge—currently in planning—is presented, highlighting its expanded focus across further clinical needs.

## 1. Introduction

**Figure 1:**
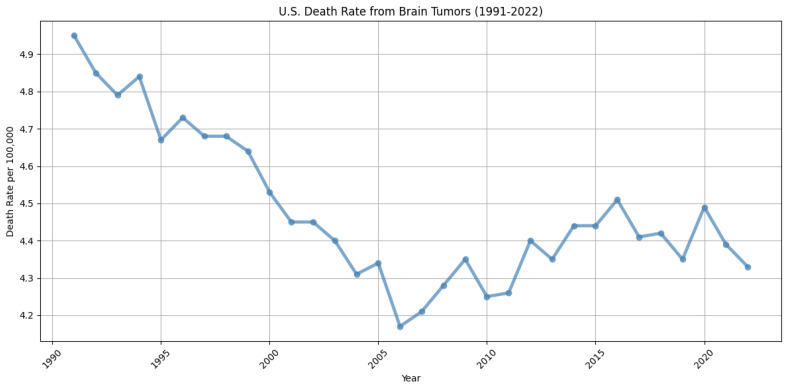
Trends in brain tumor and other nervous system cancer mortality rates in the U.S. from 1991 to 2022, derived from data in [1]. The figure shows a generally declining trend throughout the 1990s, followed by fluctuations and a near plateau in more recent years, indicating some progress, yet highlighting the continued need for innovations in diagnosis and treatment to further reduce mortality.

Brain tumors are among the most lethal forms of cancer. Notably, glioblastoma and diffuse astrocytic glioma with molecular features of glioblastoma (WHO Grade 4 astrocytoma) stand out as the most prevalent and aggressive malignant primary tumors of the central nervous system in adults. These tumors are marked by profound intrinsic heterogeneity in terms of appearance, shape, and histology, and they typically have a median survival rate of about 15 months. The diagnosis and treatment of brain tumors present significant complexities and challenges. They are notably resistant to standard therapies, partly because of the challenges in drug delivery to the brain and the extensive heterogeneity observed in their radiographic, morphological, and molecular profiles. Nevertheless, decades of intensive research have resulted in a 12.5% reduction in brain and other nervous system cancer mortality rates from 1991 to 2022 in the U.S., underscoring the critical need for ongoing advancements in the diagnosis, characterization, and treatment of these conditions [1]. The trend in death rates over the past three decades is illustrated in Figure 1, showing a marked decline during the 1990s but relative stability in more recent years. This plateau underscores the continued need for ongoing research and innovation to achieve further improvements in diagnosing and treating brain tumors.

Brain tumor segmentation plays a crucial role in medical imaging, serving as a cornerstone for accurate diagnosis, effective treatment planning, and continuous monitoring of patients with brain tumors. Magnetic resonance imaging (MRI), as a non-invasive and highly detailed imaging modality, has become the gold standard for visualizing brain structures and pathologies. Its ability to provide high-resolution, multi-contrast images of soft tissues makes it uniquely suited for identifying and delineating abnormalities, including brain tumors. However, the complexity and heterogeneity of the appearance of brain tumors, coupled with the intricate structure of the human brain, present significant challenges for manual segmentation. In addition, manually segmenting brain tumors from magnetic resonance (MR) images is an extremely time-consuming process. Moreover, human subjectivity and artifacts introduced during the imaging process can further complicate the interpretation. As a result, manual brain MRI segmentation is susceptible to both inter- and intra-observer variability.

One of the key advancements in brain tumor segmentation is the ability to delineate tumors into specific areas such as the necrotic core (NCR), enhancing tumor (ET), and peritumoral edema (ED). This fine-grained approach is critical for understanding the biological behavior of tumors, guiding surgical resections, and tailoring radiotherapy and chemotherapy protocols. The Brain Tumor Segmentation (BraTS) challenges have played a crucial role in standardizing the segmentation of these sub-regions. This standardization has facilitated the development and benchmarking of automated algorithms, specifically designed to tackle the unique complexities of gliomas and other brain tumors. Machine learning and deep learning methods have revolutionized the field, enabling automated and precise delineation of these sub-regions. The success of these methods hinges on the availability of high-quality datasets that reflect the complexity and diversity of real-world clinical scenarios. Datasets serve as the foundation for developing, training, and benchmarking segmentation algorithms. In the context of MRI-based brain tumor analysis, these datasets must offer rich, annotated data that capture the variability in tumor size, shape, and location, as well as differences in scanner types [2,3].

This paper provides a comprehensive review of the Brain Tumor Segmentation (BraTS) challenges and their datasets, spanning from 2012 to 2024, and offers an overview of the planned BraTS 2025 challenge. By examining the evolution of these datasets, we aim to highlight their unique features, such as expert-annotated ground truths, standardized imaging protocols, and incorporation of diverse clinical contexts, including post-treatment scenarios and underrepresented populations. Unlike previous works that have focused on either a single challenge year or a narrower scope, this review merges all publicly available BraTS datasets from its inception to the most recent edition, establishing a uniquely comprehensive perspective on both dataset progression and the algorithmic milestones achieved over time. Crucially, this review provides new insights into how the iterative changes in dataset size, annotation standards, and inclusion criteria are shaping the latest approaches to clinically oriented segmentation tasks, thereby guiding future research toward more robust and generalizable MRI-based segmentation methodologies. This review highlights the transformative impact of the BraTS challenges, emphasizing how the massive evolution of these datasets has driven advancements in segmentation algorithms and contributed to the development of applications with significant potential in clinical practice. By articulating these novel contributions and emphasizing gaps that remain to be explored, we position this review as a key resource for researchers aiming to push the boundaries of precision medicine through high-quality, large-scale MRI datasets.

## 2. MRI Technology: A Critical Tool for Brain Tumor Segmentation

MRI technology stands out as the only imaging modality that offers such comprehensive and detailed visualization capabilities for brain tumor segmentation. Its unique ability to provide high-contrast images of soft tissues makes it indispensable in neuro-oncology. The technical aspects of MRI, especially the use of its four primary modalities—T1, T2, T1 with contrast (T1C), and fluid-attenuated inversion recovery (FLAIR)—are essential for effective segmentation and analysis of brain tumors. Each modality offers unique information about the brain’s anatomy and pathology, enhancing the accuracy of tumor detection and characterization.

**Figure 2:**
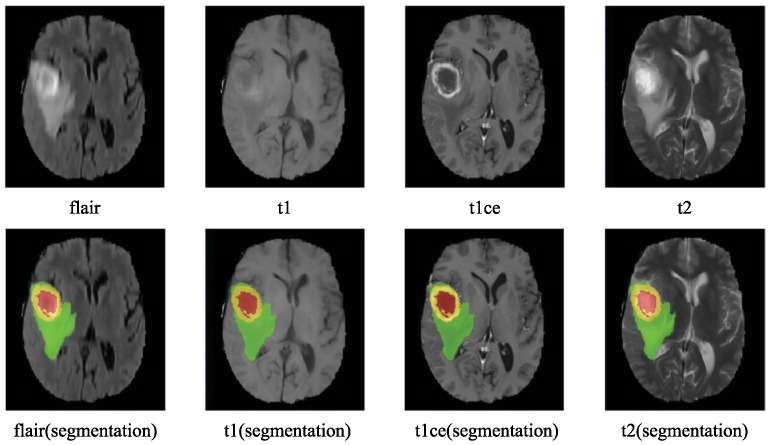
Image illustrating the four MRI modalities (T1, T2, T1C, FLAIR) alongside their corresponding ground truth (GT) segmentations. T1 emphasizes structural detail, T2 highlights fluid and edema, T1C reveals enhancing tumor regions by showing areas where the blood–brain barrier is disrupted, and FLAIR better delineates peritumoral edema by suppressing cerebrospinal fluid signals. Together, these modalities provide a comprehensive view of the tumor’s morphology and pathology, underscoring their critical role in accurate segmentation and informed treatment planning. Figure adapted from [4], licensed under the CC BY 4.0: https://creativecommons.org/licenses/by/4.0/ (accessed on 15 November 2024).

T1-weighted images help define the anatomical structure of the brain, T2-weighted images highlight fluid and edema, and T1C enhances the visualization of active tumor regions by highlighting areas where the blood–brain barrier is disrupted. FLAIR images are particularly useful in suppressing the effects of cerebrospinal fluid in the brain to better delineate the peritumoral edema and infiltrative tumor edges. As illustrated in Figure 2, these four modalities and their corresponding ground truth (GT) segmentations reveal distinct, yet complementary tumor features, providing a multi-faceted perspective. These modalities collectively provide a comprehensive view that is critical for diagnosing brain tumors, planning treatment, and monitoring progression or response to therapy.

The integration of these MRI modalities into segmentation tasks enables the development of algorithms that can accurately reflect the complex reality of brain tumor pathology, thus supporting advanced diagnostic and therapeutic strategies in clinical practice. The precision and depth of MRI data directly support the goals of precision medicine by enabling tailored treatment plans based on detailed, individualized tumor characteristics [5,6].

### Evolution of MRI Extraction Technologies from 2012 to Present

The evolution of MRI extraction technologies over the last decade has been marked by significant technological advancements that have expanded both the capabilities and the clinical utility of MRI systems. Since 2012, there have been transformative changes in how MRI data are acquired, processed, and utilized, driven by advancements in both hardware and software technologies.

Early MRI systems were primarily focused on structural imaging, with a limited ability to capture the dynamic processes of the human body. However, recent years have seen a substantial improvement in both the spatial and temporal resolution of MRI scans. This is largely due to the advent of high-density phased array coils and the implementation of parallel imaging techniques, which have drastically reduced scan times and improved image quality. These advances have been crucial in the development of functional MRI (fMRI) and diffusion tensor imaging (DTI), which provide insights into brain activity and neural connectivity. Moreover, the integration of machine learning algorithms, particularly deep learning, has revolutionized MRI data processing. These technologies enable more accurate and faster image reconstruction and have been pivotal in developing applications such as real-time imaging and automated anomaly detection. The utilization of artificial intelligence in MRI systems not only enhances the detection and characterization of pathologies but also optimizes the MRI workflow, reducing the dependency on operator expertise and improving diagnostic accuracy. The adoption of transformers in medical imaging has further enhanced capabilities, particularly in the area of feature extraction, by enabling the analysis of large data sequences with attention mechanisms that focus on relevant parts of the data, leading to more precise and efficient diagnosis.

Another notable advancement has been in the field of ultra-high-field MRI systems, which operate at field strengths of 7 Tesla or higher. These systems offer unparalleled image resolution and contrast, making them exceptionally useful for detailed anatomical studies and complex clinical research projects. The increased signal-to-noise ratio provided by these high-field systems allows for more detailed visualization of minute anatomical structures and pathological changes, which is critical for early diagnosis and treatment planning.

The period from 2012 to the present has also seen efforts to standardize MRI protocols and techniques across different centers and platforms. This standardization is crucial for multi-center studies and broad clinical applications, ensuring that MRI results are consistent and comparable regardless of where or how the data were obtained.

Recent developments hint at a future where the Mamba architecture could play a pivotal role, potentially overtaking transformers as the state-of-the-art due to its innovative approach to handling sequential data. Mamba is designed to optimize the processing of time-series data, which is particularly relevant in medical imaging where changes over time can be critical to accurate diagnoses. Unlike traditional models, Mamba incorporates mechanisms that can more effectively capture temporal dependencies and variations within the data, providing a more dynamic and responsive analysis. Furthermore, Mamba’s potential to integrate with existing deep learning frameworks offers a promising avenue for refining diagnostic tools and treatment planning systems, thereby pushing the boundaries of medical imaging technology with more precise and actionable insights [7].

In conclusion, the evolution of MRI extraction technologies over the past decade has not only enhanced the technical capabilities of MRI systems but also significantly broadened their clinical applications, making MRI an indispensable tool in modern medicine. These advancements underscore the importance of continual investment in MRI technology development, aiming for even greater accuracy, efficiency, and applicability in the years to come [8,9].

## 3. Overview of BraTS Challenges

Precise delineation of brain tumor sub-regions in MRI scans is critically important for numerous clinical practices, including planning surgical treatments, guiding image-based interventions, tracking tumor progression, and creating maps for radiotherapy [3]. Additionally, accurate segmentation of these tumor sub-regions is increasingly recognized as a foundation for quantitative image analysis that can predict overall patient survival [2].

However, manual identification and delineation of tumor sub-regions is a labor-intensive, time-consuming, and subjective task. In clinical settings, this process is typically manually performed by radiologists through qualitative visual assessment, making it impractical for handling large patient volumes. This underscores the pressing need for automated, reliable segmentation solutions to streamline and accelerate the process [3].

Since its launch in 2012, the Brain Tumor Segmentation (BraTS) challenge has been dedicated to assessing advanced methods for the segmentation of brain tumors from mpMRI scans. Initially, BraTS aimed to provide a public dataset and establish a community benchmark. It primarily utilized pre-operative mpMRI scans sourced from various institutions, focusing on the segmentation of brain tumors, especially gliomas, which exhibit extensive variability in appearance, shape, and histology. Moreover, between 2017 and 2020, BraTS expanded its scope to include predictions of patient overall survival (OS) for glioblastoma cases following gross total resection. This expansion allowed for the integration of radiomic feature analysis and machine learning algorithms to highlight the clinical importance of the segmentation task [2,3].

In the 2021–2022 iterations, BraTS continued to concentrate on segmenting glioma sub-regions, supported by a substantially increased dataset. The 2021 challenge also introduced the critical clinical task of determining the methylation status of the O6-methylguanine-DNA methyltransferase (MGMT) promoter in tumors, classifying them as either methylated or unmethylated [3].

BraTS 2023 further refined its benchmarking framework and expanded its dataset to encompass a wider patient demographic, including regions like sub-Saharan Africa, various tumor types such as meningiomas, and new clinical challenges like handling incomplete data. This edition introduced several sub-challenges addressing specific clinical needs and technical issues, such as data augmentation [10].

The 2024 edition of BraTS extended its research scope further by introducing new sub-challenges, including those focusing on post-treatment glioma outcomes. The Pathology challenge specifically aims to develop deep learning models capable of identifying distinct histological features within tumor sub-regions, thus enhancing the accuracy of brain tumor diagnosis and grading [11].

Alongside the data, the classes used for segmentation have also seen significant changes to better capture the nuances of tumor behavior and the need for more effective algorithm training. Initially, through 2016, tumor sub-regions were classified into four distinct categories. However, from 2017 onwards, the segmentation was streamlined to encompass just three categories. The editions of 2023 and 2024 further diversified the annotations and classes used, tailoring them to specific datasets and tasks, thereby adding layers of complexity to the segmentation challenges [2].

## 4. Evolution of BraTS Datasets (2012–2025)

This section explores the development and diversification of the Brain Tumor Segmentation (BraTS) challenge datasets from its inception in 2012 up to 2025. Initially focused on the segmentation of glioma sub-regions, the scope of the BraTS challenges has significantly expanded to include various brain conditions and an increased complexity in the tasks. We will examine how these datasets have evolved over time, with a particular focus on the notable shift in the variety of the challenges introduced in recent years. This evolution is documented in detail in Table 1, which provides an overview of the datasets employed each year and the specific tasks associated with them. Notably, the table shows how each BraTS edition gradually increased dataset size and diversity, culminating in more advanced tasks from 2023 onward (e.g., post-treatment imaging, synthesis challenges, meningioma segmentation). By capturing these incremental changes in case numbers, task complexity, and clinical timepoints, it provides researchers with valuable insights into the evolution of the datasets—informing the design of future segmentation models and guiding research strategies in neuro-oncology. The recent expansions reflect broader clinical applications and the integration of advanced computational techniques in the medical imaging field, necessitating more elaborate segmentation models. The latest contests are not only more complex but also occupy a significantly larger portion of the discussion due to their increased relevance and the broadened scope of the challenge.

### 4.1. Dataset Review: BraTS Challenge 2012

**Number of tasks:** 1: Multimodal Brain Tumor Image Segmentation—segmentation of gliomas in pre-operative scans.
**Initial number of classes:** two class labels:
  - Label 1—ED: edema;
  - Label 2—TC: tumor core.
**Final number of classes:** four class labels:
  - Label 1—NCR: necrotic tumor;
  - Label 2—ED: peritumoral edema;
  - Label 3—NET: non-enhancing tumor;
  - Label 4—ET: enhancing tumor;
  - Label 0: everything else.

**MRI modalities:** All four modalities were available: T1-weighted, native image (T1); T1-weighted, contrast-enhanced (Gadolinium) image (T1c); T2-weighted image (T2); and T2-weighted FLAIR image (FLAIR). In subsequent BraTS challenges, these four modalities were consistently employed unless noted otherwise in the challenge description.

**Challenge Data:** The BraTS 2012 Challenge on Multimodal Brain Tumor Image Segmentation was designed to advance the state-of-the-art methods in the segmentation of brain tumors, specifically gliomas, from multimodal MRI images. The goal was to create a benchmark and encourage the development of innovative segmentation algorithms that could efficiently navigate the complexities found in tumor imaging. By offering a platform for a detailed comparison using a standardized collection of both authentic and synthetic MRI scans, they endeavored to improve the precision and clinical utility of these technologies in neuro-oncology.

All participants received training data consisting of multi-contrast MRI scans from 10 low-grade and 20 high-grade glioma patients, originally manually annotated with two tumor labels—“Edema” and “Core”—by a trained expert. Additionally, the training set included simulated images for 25 high-grade and 25 low-grade glioma subjects labeled with the same two ground truth categories. The test set included 11 high-grade and 4 low-grade real cases, as well as 10 high-grade and 5 low-grade simulated cases [12,13].

**Figure 3:**
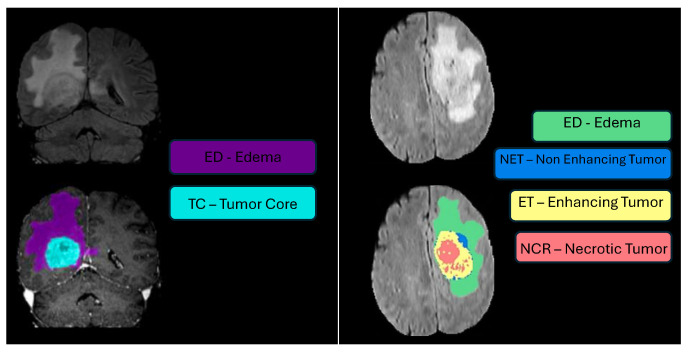
This figure illustrates the segmentation labeling used in BraTS 2012. The (**left image**) shows the initial two classes for the challenge—edema (ED) and tumor core (TC)—while the (**right image**) demonstrates the subsequent relabeling into four more specific classes: edema (ED), necrotic tumor (NCR), non-enhancing tumor (NET), and enhancing tumor (ET). By refining the broad “core” label, this updated annotation better reflects the distinct substructures observed in different MRI modalities, ultimately improving the clinical relevance. Figure adapted and modified from [14,15].

During subsequent discussions, it became clear that categorizing tumor sub-regions into two types was overly simplistic. Specifically, the category labeled “core” was found to include substructures that appeared differently across various imaging modalities. They, therefore, had the training data re-annotated with four distinct tumor labels, as illustrated in Figure 3, where the initial two classes, edema (ED) and tumor core (TC), were refined to edema (ED), necrotic tumor (NCR), non-enhancing tumor (NET), and enhancing tumor (ET). The 30 training cases were re-labeled by four different raters, and the 2012 test set was annotated by three. For datasets with multiple annotations, they fused the resulting label maps by assuming increasing ‘severity’ of the disease from edema to non-enhancing (solid) core to necrotic (or fluid-filled) core to enhancing core, using a hierarchical majority voting scheme that assigns a voxel to the highest class to which at least half of the raters agree. The synthetic images were generated using TumorSim software, which uses physical and statistical models to produce MRI images that emulate real-life tumor scenarios. These simulations were conducted with the same modalities and 1 mm resolution as the real data, using a tumor growth model to simulate infiltrating edema, local tissue distortion, and central contrast enhancement, effectively mimicking the texture of actual MRI scans.

However, in evaluating the performance of segmentation algorithms, they organized the different structures into three mutually inclusive tumor regions, which better reflect clinical tasks, such as tumor volumetry [13], and obtained the following:

1. Whole tumor region (WT): Includes all four tumor structures, corresponding to labels 1 + 2 + 3 + 4.
2. Tumor core region (TC): Contains all tumor structures except for “edema”, corresponding to labels 1 + 3 + 4.
3. Active tumor region (AT): Includes only the “enhancing core” structures that are unique to high-grade cases, corresponding to labels 1 + 4.

**Pre-Processing Data Protocol:** To ensure uniformity across the dataset, the volumetric images of each subject were rigidly aligned to their respective T1c MRI scans. Subsequently, these images were resampled to a standard resolution of 1mm³ using linear interpolation and reoriented to a consistent axial plane. The alignment was facilitated by a rigid registration model that utilized mutual information as the similarity measure, specifically using the “VersorRigid3DTransform” with the “MattesMutualInformation” metric and implemented across three multi-resolution levels within The Insight Toolkit (ITK) software. No attempt was made to align the individual patients within a common reference space. Additionally, all images underwent skull stripping to ensure the anonymization of patient data [13].
**Annotation Method:** The simulated images were provided with predefined “ground truth” data for the location of various tumor structures, whereas the clinical images were labeled manually. They established four categories of tumor sub-regions: “edema”, “non-enhancing (solid) core”, “necrotic (or fluid-filled) core”, and “enhancing core”. The annotation protocol for identifying these visual structures in both low- and high-grade cases was as follows:
  1. The “edema” was primarily segmented from T2-weighted images. FLAIR sequences were used to verify the extent of the edema and to differentiate it from ventricles and other fluid-filled structures. Initial segmentation in T2 and FLAIR included the core structures, which were then reclassified in subsequent steps.
  2. The gross tumor core, encompassing both enhancing and non-enhancing structures, was initially segmented by assessing hyper-intensities on T1c images (for high-grade tumors) along with inhomogeneous components of the hyper-intense lesion evident in T1 and the hypo-intense areas seen in T1.
  3. The “enhancing core” of the tumor was subsequently segmented by thresholding T1c intensities within the resulting gross tumor core. This segmentation included the Gadolinium-enhancing tumor rim while excluding the necrotic center and blood vessels. The intensity threshold for segmentation was determined visually for each case.
  4. The “necrotic (or fluid-filled) core” was identified as irregular, low-intensity necrotic areas within the enhancing rim on T1c images. This label was also applied to the occasional hemorrhages observed in the BRATS dataset.
  5. The “non-enhancing (solid) core” was characterized as the part of the gross tumor core remaining after the exclusion of the “enhancing core” and the “necrotic (or fluid-filled) core”.

Following this method, MRI scans were annotated by a combined team of seven radiologists and radiographers, distributed across Bern, Debrecen, and Boston. They marked structures on every third axial slice, used morphological operators for interpolation (e.g., region growing), and conducted visual inspections to make any necessary manual corrections. All segmentations were carried out using 3D Slicer software, with each subject taking approximately 60 min to process.

**Multi-Center Imaging Data Acquisition Details:** The data were collected from four distinct institutions: Bern University, Debrecen University, Heidelberg University, and Massachusetts General Hospital. These images were gathered over several years, utilizing MRI scanners from various manufacturers, featuring varying magnetic field strengths (1.5 T and 3 T) and different implementations of imaging protocols (such as 2D or 3D) [12,13].

### 4.2. Dataset Review: BraTS Challenge 2013

**Number of tasks:** 1: Multimodal Brain Tumor Image Segmentation—segmentation of gliomas in pre-operative scans.
**Number of classes:** four class labels:
  - Label 1—NCR: necrotic tumor;
  - Label 2—ED: peritumoral edema;
  - Label 3—NET: non-enhancing tumor;
  - Label 4—ET: enhancing tumor;
  - Label 0: everything else.

**Challenge Data:** BraTs 2013 had the same objective as BraTS 2012. The training data for BraTS 2013 was identical to the real training data of the 2012 Challenge but with the updated labels (4-class labels). No synthetic cases were evaluated in 2013, and therefore, no synthetic training data were provided. In addition, 10 new data were added to the test set. To summarize, the training data consisted of 30 multi-contrast MRI scans of 10 low- and 20 high-grade glioma patients, while the test images consisted of 25 MRI scans of 11 high- and 4 low-grade real cases from BraTS 2012, as well as 10 new high-grade real cases. Note that the pre-processing Data Protocol, the Annotation Method, and the contributing institutions (Multi-Center Imaging Data Acquisition Details) are the same as in BraTS 2012 [13].

### 4.3. Dataset Review: BraTS Challenge 2014–2016

**Number of tasks:** 2:
  1. Multimodal Brain Tumor Image Segmentation—segmentation of gliomas in pre-operative scans;
  2. Disease progression assessment.
**Number of classes:** four class labels:
  - Label 1—NCR: necrotic tumor;
  - Label 2—ED: peritumoral edema;
  - Label 3—NET: non-enhancing tumor;
  - Label 4—ET: enhancing tumor;
  - Label 0: everything else.

**Challenge Data:** BraTS has predominantly been engaged in the segmentation of brain tumor sub-regions. Yet, beyond its initial editions in 2012–2013, the challenge’s potential for clinical impact became evident. Subsequently, BraTS introduced secondary tasks, leveraging the outcomes of brain tumor segmentation algorithms to enhance further analyses. Particularly, to highlight the clinical relevance of these segmentation tasks, the BraTS challenges from 2014 to 2016 incorporated longitudinal scans into the datasets. These additions aimed to assess the efficacy and potential of automated tumor volumetry in monitoring disease progression [2].

For the years 2014 to 2016, the dataset was an expanded version of the one used in the 2013 challenge, featuring 300 high-grade (HG) and low-grade (LG) glioma brain scans from the NIH Cancer Imaging Archive (TCIA). Segmentation was performed both manually and algorithmically, following the same classification system of the years before. The ground truth annotation was derived by the fusion of segmentation results from top-performing algorithms from the 2012 and 2013 BraTS challenges, which were subsequently validated through visual inspections by experienced raters. Moreover, the 2014 challenge introduced a dataset comprising longitudinal series of scans for individual patients aimed at assessing disease progression. This dataset included 25 cases, each documented at 3 to 10 different time points. While training dataset annotations in some challenges were automated, the annotation of the test dataset was always performed manually by expert raters in all BraTS challenges [16]. Variations in the datasets from BraTS 2014, 2015, and 2016 are detailed in Table 2. Due to the algorithmic assessment of the ground truth (GT), datasets from these years were subsequently discarded and manually reannotated by experts in the following years.

**Annotation Method:** The annotation was accomplished by fusing results of high-ranked segmentation algorithms in BraTS 2012 and BraTS 2013 challenges. These annotations were then approved by visual inspection of experienced raters [16].

### 4.4. Dataset Review: BraTS Challenge 2017

**Number of tasks:** 2:
  1. Multimodal Brain Tumor Image Segmentation—segmentation of gliomas in pre-operative scans;
  2. Prediction of patient overall survival (OS) from pre-operative scans.
**Number of classes:** three class labels:
  - Label 1—NCR/NET: necrotic and non-enhancing tumor;
  - Label 2—ED: the peritumoral edema;
  - Label 4—ET: the GD-enhancing tumor;
  - Label 0: everything else.

**Figure 4:**
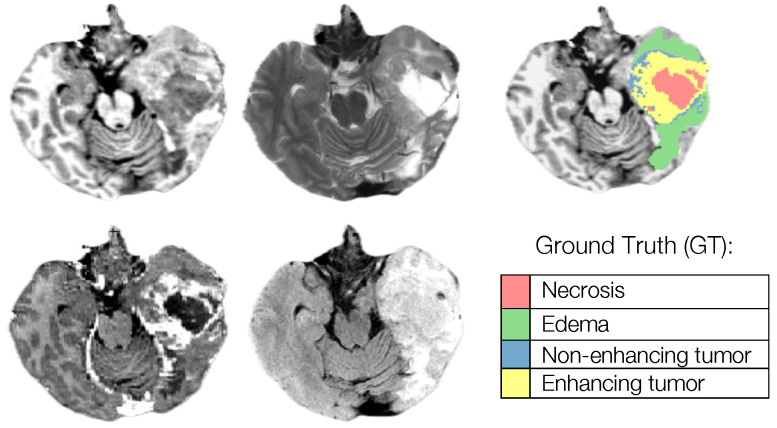
Example highlighting the limited imaging evidence for Label 3 (non-enhancing tumor), as seen in the overlaid ground-truth segmentation. The subtlety of the non-enhancing region can lead to potential overestimation and variability in annotations across different institutions. Figure adapted and modified from https://academictorrents.com/details/c4f39a0a8e46e8d2174b8a8a81b9887150f44d50. Originally published under the CC BY-NC-SA 3.0: https://creativecommons.org/licenses/by-nc-sa/3.0/it/deed.en (accessed on 15 September 2024).

Experts noted that the NET (non-enhancing tumor, labeled as ‘Label 3’) can sometimes be overestimated by annotators, with limited evidence available in the imaging data to clearly define this sub-region. Figure 4 illustrates a sample slice where the non-enhancing tumor region is ambiguous; the overlaid ground truth segmentation reveals how such labeling can be influenced by minimal or uncertain imaging signals. Imposing labels where the imaging data do not clearly support them can lead to inconsistencies, resulting in ground truth labels that vary widely among different institutions. Such discrepancies could influence the rankings of BraTS participants, skewing results in favor of an annotator’s interpretation over the actual efficacy of the segmentation algorithms. To solve this problem, starting in 2017, the BraTS challenge removed the NET label, combining it with the necrotic core (NCR, labeled as ‘Label 1’). This adjustment was made to simplify annotations and enhance the consistency of algorithm evaluations across different datasets.

Additionally, areas of T2-FLAIR hyper-intensity in contralateral and periventricular regions were no longer included in the edema (ED) region, unless directly adjacent to peritumoral ED. These areas are often indicative of chronic microvascular alterations or age-related demyelination rather than active tumor infiltration [2].

**Figure 5:**
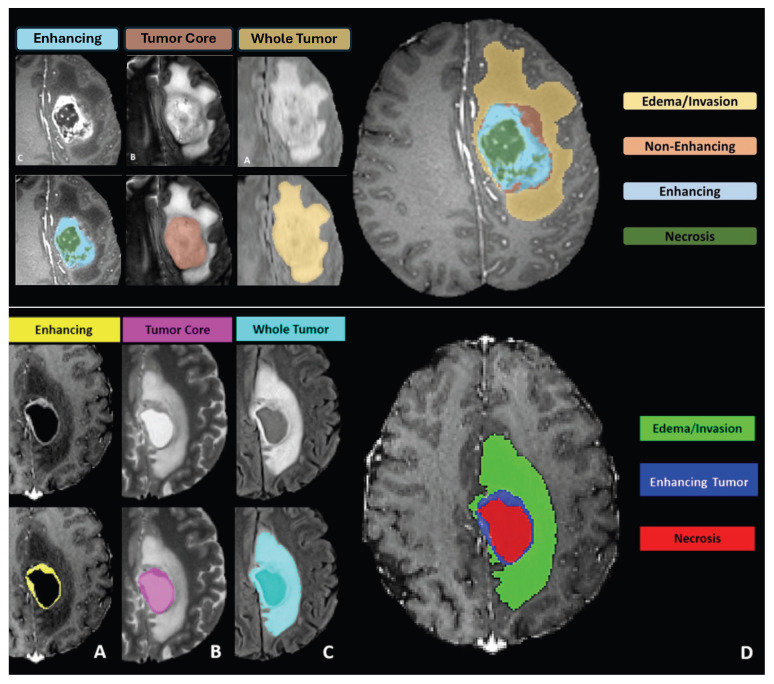
At the top, the image presents the four-class labeling system used for BraTS challenges up to 2017, including edema (ED), necrotic tumor (NCR), non-enhancing tumor (NET), and enhancing tumor (ET). Below, the image shows the revised four-class labeling system introduced in 2017, which merges NET with NCR to reduce annotation ambiguity and improve consistency. Both the top and bottom images depict the labeling system from left to right: Panel (**A**) shows the enhancing tumor structures visible in a T1c scan surrounding the cystic/necrotic components of the core; Panel (**B**) displays the tumor core visible in a T2 scan; Panel (**C**) illustrates the whole tumor visible in a FLAIR scan; and Panel (**D**) depicts the combined segmentations generating the final tumor sub-region labels. These panels apply to both the original and revised three-class labeling systems shown in the image. This streamlined labeling approach underpinned the BraTS 2017 segmentation tasks and facilitated more reliable comparisons of algorithmic performance. Figure adapted and modified from [3], licensed under the CC BY 4.0: https://creativecommons.org/licenses/by/4.0/, and [13], licensed under the CC BY-NC-SA 4.0: https://creativecommons.org/licenses/by-nc-sa/4.0/ (accessed on 15 September 2024).

**Challenge Data:** Task 1 involved developing methodologies for segmenting gliomas on pre-operative scans, utilizing clinically-acquired training data. The analyzed sub-regions included: (1) the enhancing tumor (ET), (2) the tumor core (TC), and (3) the whole tumor (WT). Specifically, WT is defined as the combination of labels 1, 2, and 4; TC comprises labels 1 and 4; and ET corresponds solely to label 4. Figure 5 illustrates how the previous four-class labeling (top panel) was refined in 2017 by omitting the non-enhancing tumor label (NET) and merging it with the necrotic core (NCR). This shift simplified annotations and ensured greater consistency in segmentation tasks across diverse data sources.

After generating the segmentation labels on the pre-operative scans for Task 1, participants proceeded to Task 2. Here, they leveraged these labels along with provided multimodal MRI data to identify and analyze appropriate imaging/radiomic features using machine learning algorithms, aiming to predict patient overall survival (OS).

The dataset provided in BraTS 2017 differed significantly from the datasets used in previous challenges from 2014 to 2016. Crucially, the data from these years, sourced from TCIA and containing a mix of pre- and post-operative scans, were discarded not only because they included post-operative cases but mainly due to concerns regarding the reliability of their ground truth labels. These labels were generated by merging results from algorithms that performed well in the BraTS 2012 and 2013 challenges, rather than from manual expert annotations. This raised issues about the consistency and accuracy of these labels. For BraTS 2017 and subsequent challenges, only data from BraTS 2012–2013, which had been manually annotated by clinical experts, were retained. The datasets from BraTS 2014–2016 were excluded to ensure the ground truth labels used going forward were derived from expert manual annotations. All pre-operative scans from the TCIA collections (TCGA-GBM, n = 262 and TCGA-LGG, n = 199) were reassessed by board-certified neuroradiologists who then annotated the identified pre-operative scans (135 GBM and 108 LGG) for various glioma sub-regions [17]. Detailed procedures for these annotations are outlined in the referenced paper [18].

In 2017, BraTS introduced the new challenge of predicting patient overall survival (OS) based on pre-operative MRI scans from patients who had undergone gross total resection. For this task, the survival data (in days) for 163 training cases were provided. Participants used their segmentation predictions to extract relevant radiomic features from the MRI scans, which were then processed through machine-learning models to predict OS [16].

**Pre-Processing Data Protocol:** A consistent pre-processing protocol has been applied to all BraTS mpMRI scans. This protocol involves converting DICOM files to the NIfTI format, co-registering images to a standard anatomical template (SRI24), resampling them to a uniform isotropic resolution (1 ${\mathrm{mm}}^{3}$), and performing skull-stripping. Detailed information on the entire pre-processing pipeline is available through the Cancer Imaging Phenomics Toolkit (CaPTk) and the Federated Tumor Segmentation (FeTS) tool. Converting to NIfTI format removes accompanying metadata from the original DICOM images, effectively stripping out all Protected Health Information (PHI) from the DICOM headers. Additionally, skull-stripping helps prevent any potential facial reconstruction or patient identification. Note that in subsequent challenges, this standardized pre-processing protocol has been consistently employed unless otherwise specified in the challenge description.
**Annotation Method:** All ground truth labels from BraTS 2016 were meticulously revised by expert board-certified neuroradiologists. Each imaging dataset underwent manual segmentation by one to four raters, adhering to a consistent annotation protocol. These annotations, which include the GD-enhancing tumor (ET—label 4), the peritumoral edema (ED—label 2), and the necrotic and non-enhancing tumor (NCR/NET—label 1), received approval from experienced neuroradiologists. For further details on the pre-processing and annotation protocols, refer to the following papers: [2,13,18].
**Multi-Center Imaging Data Acquisition Details:** The multimodal MRI data were acquired with different clinical protocols and various scanners from multiple (n = 19) institutions. The data contributors are located in the following countries: United States, Switzerland, Hungary, and Germany.

### 4.5. Dataset Review: BraTS Challenge 2018

**Number of tasks:** 2:
  1. Multimodal Brain Tumor Image Segmentation—segmentation of gliomas in pre-operative scans;
  2. Prediction of patient overall survival (OS) from pre-operative scans.
**Number of classes:** three class labels:
  - Label 1—NCR/NET: necrotic and non-enhancing tumor;
  - Label 2—ED: the peritumoral edema;
  - Label 4—ET: the GD-enhancing tumor;
  - Label 0: everything else.

**Challenge Data:** The tasks for BraTS 2018 remained unchanged from BraTS 2017: participants were required to develop methods for generating segmentation labels for various glioma sub-regions and to extract imaging/radiomic features to predict patient overall survival (OS) using machine learning techniques. The dataset for BraTS 2018 utilized the same training set as in 2017, but featured different validation and test sets. All ground truth labels were carefully revised by expert board-certified neuroradiologists, and the validation set’s ground truth was not disclosed to participants [16,19].
It is important to note that only patients who underwent Gross Total Resection (GTR) were considered for the OS prediction analyses [19].
**Annotation Method:** All the imaging datasets underwent manual segmentation by one to four raters, following a consistent annotation protocol. All annotations received approval from experienced neuroradiologists. For additional information on the pre-processing and annotation protocols, refer to these papers: [2,13,18].
**Multi-Center Imaging Data Acquisition Details:** All BraTS multimodal scans were acquired with different clinical protocols and various scanners from multiple (n = 19) institutions. The institutions contributing to the data are located in the following countries: United States, Switzerland, Hungary, Germany, and India.

### 4.6. Dataset Review: BraTS Challenge 2019

**Number of tasks:** 3:
  1. Multimodal Brain Tumor Image Segmentation—segmentation of gliomas in pre-operative scans;
  2. Prediction of patient overall survival (OS) from pre-operative scans;
  3. Quantification of Uncertainty in Segmentation.
**Number of classes:** three class labels:
  - Label 1—NCR/NET: necrotic and non-enhancing tumor;
  - Label 2—ED: the peritumoral edema;
  - Label 4—ET: the GD-enhancing tumor;
  - label 0: everything else.

**Challenge Data:** In addition to Tasks 1 and 2 from BraTS 2018 and 2017, BraTS 2019 introduced a new challenge focused on evaluating uncertainty measures in glioma segmentation. This task aimed to encourage methods that yield high confidence when predictions are accurate and low confidence when they are not. Participants were required to submit three separate uncertainty maps—one for each voxel label corresponding to: (1) enhancing tumor (ET), (2) tumor core (TC), and (3) whole tumor (WT). These maps were to be evaluated in conjunction with the established BraTS Dice metric.
The BraTS 2019 challenge provided participants with multi-institutional, routinely acquired pre-operative multimodal MRI scans of high-grade glioblastoma (GBM/HGG) and lower-grade glioma (LGG), all of which had a pathologically confirmed diagnosis and available OS data. The datasets for this year’s challenge were enhanced since BraTS 2018, incorporating more routinely acquired 3T multimodal MRI scans, each annotated with ground truth labels by expert board-certified neuroradiologists. In this challenge, as well as in all subsequent ones, the ground truth labels for the validation data were not provided to the participants. Consistent with prior iterations, only patients who had undergone a gross total resection (GTR) were eligible for the OS prediction assessment [20].
**Annotation Method:** All the imaging datasets underwent manual segmentation by one to four raters, following a consistent annotation protocol. All annotations received approval from experienced neuroradiologists. For additional information on the pre-processing and annotation protocol, refer to these papers: [2,13,18].
**Multi-Center Imaging Data Acquisition Details:** All BraTS multimodal MRI data were acquired using different clinical protocols and various scanners from multiple (n = 19) institutions. The institutions contributing to the data are located in the following countries: United States, Switzerland, Hungary, Germany, and India.

### 4.7. Dataset Review: BraTS Challenge 2020

**Number of tasks:** 3:
  1. Multimodal Brain Tumor Image Segmentation—segmentation of gliomas in pre-operative scans;
  2. Prediction of patient overall survival (OS) from pre-operative scans;
  3. Quantification of Uncertainty in Segmentation.
**Number of classes:** three class labels:
  - Label 1—NCR/NET: necrotic and non-enhancing tumor;
  - Label 2—ED: peritumoral edema;
  - Label 4—ET: GD-enhancing tumor;
  - Label 0: everything else.

**Challenge Data:** For this year’s BraTS challenge, the dataset was enhanced with an increased number of routinely acquired 3T multimodal MRI scans, all annotated with ground truth labels by expert board-certified neuroradiologists. The format of the overall survival (OS) data remained consistent with previous years. Moreover, to aid in broader research efforts, they have provided a naming convention and direct filename mapping between the data from BraT 2020 to 2017 and the TCGA-GBM and TCGA-LGG collections hosted by The Cancer Imaging Archive (TCIA), thereby supporting studies that extend beyond the immediate scope of the BraTS tasks [21].
**Annotation Method:** All datasets underwent manual segmentation by one to four raters, adhering to a consistent annotation protocol. All annotations received approval from experienced neuroradiologists. For additional information on the pre-processing and annotation protocols, refer to these papers: [2,13,18].
**Multi-Center Imaging Data Acquisition Details:** All BraTS multimodal MRI data were acquired with different clinical protocols and various scanners from multiple (n = 19) institutions. The institutions contributing to the data are located in the following countries: United States, Switzerland, Hungary, Germany, and India.

### 4.8. Dataset Review: BraTS Challenge 2021

**Number of tasks:** 2:
  1. Multimodal Brain Tumor Image Segmentation—segmentation of gliomas in pre-operative scans;
  2. Radiogenomic Classification—evaluation of classification methods to predict the MGMT promoter methylation status at pre-operative baseline scans.
**Number of classes:** three class labels:
  - Label 1—NCR: necrotic tumor;
  - Label 2—ED: peritumoral edema;
  - Label 4—ET: GD-enhancing tumor;
  - Label 0: everything else.

**Challenge Data:** The BraTS Challenge 2021 was hosted by the Radiological Society of North America (RSNA), the American Society of Neuroradiology (ASNR), and the Medical Image Computing and Computer-Assisted Interventions (MICCAI) Society. The RSNA-ASNR-MICCAI BraTS 2021 challenge utilized multi-institutional multi-parametric magnetic resonance imaging (mpMRI) scans, continuing to focus on Task 1: assessing advanced methods for segmenting brain glioblastoma sub-regions in mpMRI scans.

The 2021 revision of the World Health Organization (WHO) classification of CNS tumors underscored the importance of integrated diagnostics, transitioning from solely morphologic–histopathologic classifications to incorporating molecular–cytogenetic features. One such feature, the methylation status of the O6-methylguanine-DNA methyltransferase (MGMT) promoter in newly diagnosed GBM, was recognized as a significant prognostic factor and predictor of chemotherapy response. Consequently, determining the MGMT promoter methylation status in newly diagnosed GBM became crucial for guiding treatment decisions. In response, BraTS 2021 introduced Task 2, which evaluated classification methods for predicting the MGMT promoter methylation status from pre-operative baseline scans, specifically distinguishing between methylated (MGMT+) and unmethylated (MGMT-) tumors.
The RSNA-ASNR-MICCAI BraTS 2021 Challenge released the largest and most diverse retrospective cohort of glioma patients to date. The impact of this challenge is underscored by the dramatic increase in the number of participating teams, which rose from 78 in 2020 to over 2300 in 2021. The datasets for this year’s challenge were notably expanded since BraTS 2020 with a significant increase in routine clinically-acquired mpMRI scans, raising the total number of cases from 660 to 2040 and thereby broadening the demographic diversity of the patient population represented. Ground truth annotations of the tumor sub-regions were all verified by expert neuroradiologists for Task 1, while the MGMT methylation status was determined based on laboratory assessments of the surgical brain tumor specimens [3,22].
**Pre-Processing Data Protocol:** For Task 1, which involves the segmentation of tumor sub-regions, the standardized pre-processing protocol established by BraTS has been applied to all mpMRI scans. For Task 2, focusing on radiogenomic classification, all imaging volumes were initially processed as in Task 1 to produce skull-stripped volumes. These were then reconverted from NIfTI back to the DICOM format. Once the mpMRI sequences were reverted to DICOM, further de-identification was implemented through a two-step process. For more details on the pre-processing protocols employed for both tasks, refer to this paper [3].
**Annotation Protocol:** In earlier BraTS challenges, specifically for Task 1, the annotation process typically began with manual delineation of the abnormal signal on T2-weighted images to define the WT, followed by the TC, and ultimately addressing the enhancing and non-enhancing/necrotic core, often employing semi-automatic tools. For BraTS 2021, to streamline the annotation process, initial automated segmentations were produced by combining methods from previously top-performing algorithms at BraTS. These included DeepMedic, DeepScan, and nnU-Net, all of which were trained on the BraTS 2020 dataset. The STAPLE label fusion technique was utilized to merge the segmentations from these individual methods, helping to address systematic errors from each. This entire segmentation process, including the automated fusion technique, has been made accessible on the Federated Tumor Segmentation (FeTS) platform.
Volunteer expert neuroradiology annotators were provided with all four mpMRI modalities alongside the automated fused segmentation volumes to begin manual adjustments. ITK-SNAP software was employed for these refinements. After the initial automated segmentations were refined, they were reviewed by two senior attending board-certified neuroradiologists, each with over 15 years of experience. Based on their evaluation, these segmentations were either approved or sent back to the annotators for further adjustments. This iterative process continued until the refined tumor sub-region segmentations were deemed satisfactory for public release and use in the challenge. The tumor sub-regions were delineated according to established radiological observations (VASARI features) and consist of the Gd-enhancing tumor (ET—label 4), the peritumoral edematous or invaded tissue (ED—label 2), and the necrotic tumor core (NCR—label 1) [3]. The significant annotation contributions to the dataset were made possible by over forty volunteer neuroradiology experts from around the world [22].
The determination of the MGMT promoter methylation status in the BraTS 2021 dataset at each hosting institution was performed using various methods, including pyrosequencing and next-generation quantitative bisulfite sequencing of promoter CpG sites. Adequate tumor tissue collected at the time of surgery was required for these assessments.
**Multi-Center Imaging Data Acquisition Details:** The dataset describes a collection of brain tumor mpMRI scans acquired from multiple different institutions under standard clinical conditions, but with different equipment and imaging protocols, resulting in a vastly heterogeneous image quality reflecting diverse clinical practice across different institutions [3]. The multimodal scans were collected from fourteen distinct institutions that are located in the following countries: United States, Germany, Switzerland, Canada, Hungary, and India.

### 4.9. Dataset Review: BraTS Challenge 2022

The BraTS 2022 Challenge continued the evaluation of Task 1 from BraTS 2021. These algorithms were trained on the 2021 dataset and tested on three distinct datasets: (i) the 2021 testing dataset of adult-type diffuse gliomas (n = 570), (ii) an independent multi-institutional dataset (Africa-BraTS) representing underrepresented Sub-Saharan African (SSA) patient populations with adult-type diffuse gliomas (n = 150), and (iii) an independent dataset comprising pediatric-type diffuse glioma cases (n = 95). The latter two additional test sets were included to evaluate the generalizability of the algorithms [23,24].

### 4.10. Dataset Review: BraTS Challenge 2023

**Number of tasks:** 8:
  1. Segmentation—adult glioma: RSNA-ASNR-MICCAI BraTS Continuous Evaluation Challenge;
  2. Segmentation—BraTS-Africa: BraTS Challenge on Sub-Sahara-Africa Adult Glioma;
  3. Segmentation—meningioma: ASNR-MICCAI BraTS Intracranial Meningioma Challenge;
  4. Segmentation—brain metastases: ASNR-MICCAI BraTS Brain Metastasis Challenge;
  5. Segmentation—pediatric tumors: ASNR-MICCAI BraTS Pediatrics Tumor Challenge;
  6. Synthesis (Global)—missing MRI: ASNR-MICCAI BraTS MRI Synthesis Challenge (BraSyn);
  7. Synthesis (Local)—inpainting: ASNR-MICCAI BraTS Local Synthesis of Tissue via Inpainting;
  8. Evaluating Augmentations for BraTS: BraTS Challenge on Relevant Augmentation Techniques.

**Number of classes for tasks 1–8:** three class labels:
  - Label 1—NCR: necrotic tumor core;
  - Label 2—ED: peritumoral edematous/invaded tissue;
  - Label 3—ET: GD-enhancing tumor;
  - Label 0: everything else.

It is important to note that starting this year, the label value for enhancing tumor (ET) has been updated to “3”, replacing the previous value of “4” used in earlier BraTS Challenges.

**Number of classes for tasks 2–4:** three class labels:
  - Label 1—NETC: non-ehancing tumor core;
  - Label 2—SNFH: surrounding non-enhancing FLAIR hyperintensity;
  - Label 3—ET: enhancing tumor;
  - Label 0: everything else.

**Number of classes for tasks 5:** three class labels:
  - Label 1—NC: non-enhancing component (a combination of nonenhancing tumor, cystic component, and necrosis);
  - Label 2—ED: peritumoral edematous area;
  - Label 3—ET: enhancing tumor;
  - Label 0: everything else.

**Figure 6:**
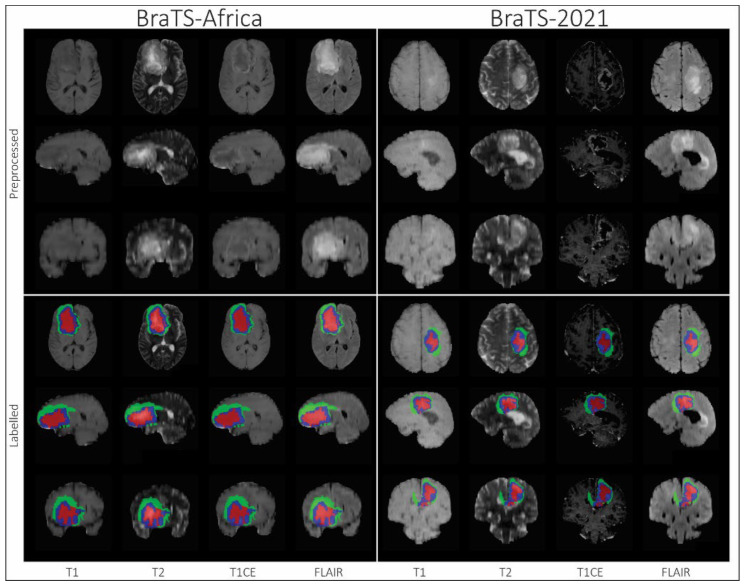
Typical clinical brain MRI obtained in Sub-Saharan Africa (**left**) compared to a representative case from the 2021 BraTS challenge (**right**). The images display notably lower resolution—especially in the sagittal plane—when compared to standard clinical imaging from high-income regions. These variations in image quality and acquisition parameters can pose additional challenges for automated algorithms. Figure adapted from [25], licensed under CC BY 4.0: https://creativecommons.org/licenses/by/4.0/ (accessed on 25 September 2024).

**Figure 7:**
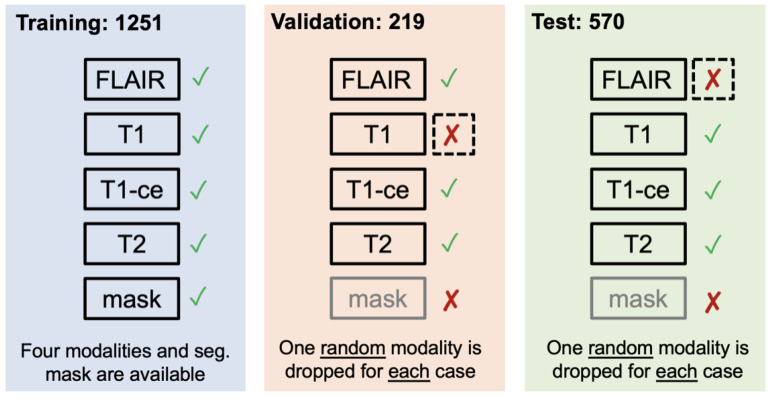
Structure of the BraSyn-2023 training, validation, and test sets. During the validation and test phases, one of the four modalities will be randomly excluded (referred to as ‘dropout’). This setup compels participants to synthesize the missing modality and maintain robust segmentation performance, reflecting real-world scenarios where complete imaging data may not always be available. Figure adapted from [28], licensed under the Creative Commons Attribution 4.0 International License: https://creativecommons.org/licenses/by/4.0/ (accessed on 25 September 2024).

**Figure 8:**
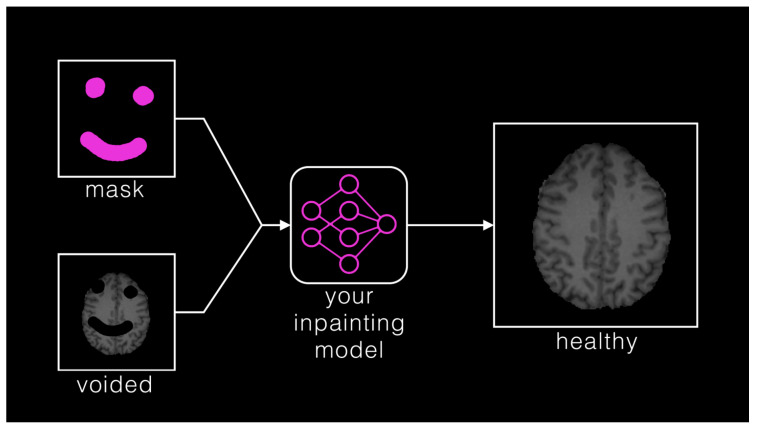
The participant’s task is to create models that synthesize healthy brain tissue within voided regions, guided by an inpainting mask, thereby reconstructing missing anatomy. This approach enables standard analysis tools to operate on tumor-affected brains by replacing pathological regions with plausible healthy tissue. Figure adapted from [29], licensed under the non-exclusive license to distribute: https://arxiv.org/licenses/nonexclusive-distrib/1.0/license.html (accessed on 25 September 2024).

**MRI modalities:** All four modalities were available: T1-weighted, native image (T1); T1-weighted, contrast-enhanced (Gadolinium) image (T1c); T2-weighted image (T2), and T2-weighted FLAIR image (FLAIR).
Primarily due to computational constraints, the synthesis (local)–inpainting task exclusively utilized T1 scans.
**Challenge Data:**
  1. Segmentation—adult glioma: RSNA-ASNR-MICCAI BraTS Continuous Evaluation Challenge: The BraTS Continuous Challenge is a continuation of the 2021 challenge. The training and validation datasets, identical to those used for the RSNA-ASNR-MICCAI BraTS 2021 Challenge, encompass a total of 5880 MRI scans from 1470 patients with brain diffuse gliomas. While the training and validation data remained consistent with those used in BraTS 2021, the testing dataset for this year’s challenge was significantly expanded to include a larger number of routine clinically-acquired mpMRI scans [10].
  2. Segmentation—BraTS-Africa: BraTS Challenge on Sub-Sahara-Africa Adult Glioma: The BraTS-Africa Challenge focuses on addressing the disparity in glioma treatment outcomes between high-income regions and Sub-Saharan Africa (SSA), where survival rates have not improved significantly due to factors such as the use of lower-quality MRI technology (see Figure 6), late-stage disease presentation, and unique glioma characteristics. Brain MRI scans from SSA typically exhibit reduced contrast and resolution, as demonstrated in Figure 6, which underscores the need for advanced pre-processing to enhance image quality prior to ML-based segmentation. This challenge is part of a broader effort to adapt and evaluate computer-aided diagnostic (CAD) tools for glioma detection in resource-limited settings, aiming to bridge the gap between research and clinical practice.
    The MICCAI-CAMERA-Lacuna Fund BraTS-Africa 2023 Challenge has assembled the largest publicly available retrospective cohort of pre-operative glioma MRI scans from adult Africans, including both low-grade glioma (LGG) and glioblastoma/high-grade glioma (GBM/HGG). The BraTS-Africa challenge involves developing machine learning algorithms to automatically segment intracranial gliomas into three distinct classes using a new 3-label system. The sub-regions for evaluation are enhancing tumor (ET), non-enhancing tumor core (NETC), and surrounding non-enhancing FLAIR hyperintensity (SNFH), which are crucial for enhancing diagnostic accuracy and treatment planning in these underserved populations [10,25].
  3. Segmentation—meningioma: ASNR-MICCAI BraTS Intracranial Meningioma Challenge: Meningioma is the most prevalent intracranial brain tumor in adults, often causing significant health issues. While about 80% of these tumors are benign WHO grade 1 meningiomas, effectively managed through observation or therapy, the more aggressive grades 2 and 3 meningiomas pose greater risks, frequently recurring despite comprehensive treatment. Currently, there is no effective noninvasive technique for determining the grade of meningioma, its aggressiveness, or for predicting outcomes.
    Automated brain MRI tumor segmentation has evolved into a clinically useful tool that provides precise measurements of tumor volume, aiding in surgical and radiotherapy planning and monitoring treatment responses. Yet, most segmentation research has primarily focused on gliomas. Meningiomas present unique challenges for segmentation due to their extra-axial location and the likelihood of involving the skull base. Moreover, since meningiomas are often identified through imaging alone, accurate MRI analysis becomes crucial for effective treatment planning.
    The aim of the BraTS 2023 Meningioma Challenge was to develop an automated multi-compartment brain MRI segmentation algorithm specifically for meningiomas. This tool was intended not only to assist in accurate surgical and radiotherapy planning but also to pave the way for future research into meningioma classification, aggressiveness evaluation, and recurrence prediction based solely on MRI scans. For this challenge, all meningioma MRI scans were taken pre-operatively and pre-treatment. The objective was to automate the segmentation of these tumors using a three-label system: enhancing tumor (ET), non-enhancing tumor core (NETC), and surrounding non-enhancing FLAIR hyperintensity (SNFH) [10].
  4. Segmentation—brain metastases: ASNR-MICCAI BraTS Brain Metastasis Challenge: Brain metastases are the most prevalent type of CNS malignancy in adults and pose significant challenges in clinical assessments. This complexity is mainly due to the common occurrence of multiple, often small, metastases within a single patient. Additionally, the extensive time required to meticulously analyze multiple lesions across consecutive scans complicates detailed evaluations. Consequently, the development of automated segmentation tools for brain metastases is vital for enhancing patient care. Precisely detecting small metastatic lesions, especially those under 5 mm, is crucial for improving patient prognosis, as overlooking even a single lesion could result in treatment delays and repeated medical procedures. Moreover, the total volume of brain metastases is a critical indicator of patient outcomes, yet it remains unmeasured in routine clinical settings due to the lack of effective volumetric segmentation tools.
    The solution lies in crafting innovative segmentation algorithms capable of identifying and accurately quantifying the volume of all lesions. While some algorithms perform well with larger metastases, they often fail to detect or accurately segment smaller metastases. The inclusion criteria for the BraTS 2023 Brain Metastases challenge were MRI scans that showed untreated brain metastases with all four MRI modalities. Exclusion criteria included scans with prior treatment effects, missing required MRI sequences, or poor-quality images due to motion or other significant artifacts. Cases with post-treatment changes were deferred to BraTS-METS 2024.
    The dataset encompasses a collection of treatment-naive brain metastases mpMRI scans from various institutions, captured under standard clinical protocols. A total of 1303 studies were annotated, with 402 studies comprising 3076 lesions made publicly available for the competition. Additionally, 31 studies with 139 lesions were set aside for validation, and 59 studies with 218 lesions were designated for testing. Notably, the Stanford University dataset, despite being publicly accessible, was excluded from the primary dataset due to the absence of T2 sequences but was available for optional additional training [10,26].
  5. Segmentation—pediatric tumors: ASNR-MICCAI BraTS Pediatrics Tumor Challenge: The ASNR-MICCAI BraTS Pediatrics Tumor 2023 Challenge marked the inaugural focus on pediatric brain tumors within the BraTS series, targeting a significant area of concern as pediatric central nervous system tumors are the leading cause of cancer-related mortality in children. The five-year survival rate for high-grade gliomas in children is less than 20%. Furthermore, due to their rarity, the diagnosis is often delayed and treatment relies on historical methods, with clinical trials requiring collaboration across multiple institutions.
    The 2023 Pediatrics Tumor Challenge leveraged a comprehensive international dataset gathered from consortia dedicated to pediatric neuro-oncology. Notably, it provided the largest annotated public retrospective cohort of high-grade pediatric glioma cases.
    The dataset for BraTS-PEDs 2023 included a multi-institutional cohort of standard clinical MRI scans, with inherent variations in imaging protocols and equipment across different institutions contributing to the diversity in image quality. The inclusion criteria for the challenge were pediatric patients with histologically confirmed high-grade gliomas, ensuring the availability of all four mpMRI sequences on treatment-naive scans. Exclusion criteria included images of poor quality or containing artifacts that hinder reliable tumor segmentation and infants younger than one month.
    Data for a total of 228 pediatric patients with high-grade gliomas was sourced from CBTN, Boston Children’s Hospital, and Yale University. This cohort was divided into subsets for training (99 cases), validation (45 cases), and testing (84 cases) [27].
  6. Synthesis (Global)—missing MRI: ASNR-MICCAI BraTS MRI Synthesis Challenge (BraSyn). Most segmentation algorithms depend on the availability of four standard MRI modalities: T1-weighted images with and without contrast, T2-weighted images, and FLAIR images. However, in clinical settings, certain sequences might be missing due to factors like time constraints or patient movement, which can introduce artifacts. Therefore, developing methods to effectively substitute missing modalities and maintain segmentation accuracy is crucial for these algorithms to be widely used in clinical routines. To address this, BraTS 2023 launched the Brain MR Image Synthesis (BraSyn) Challenge, aimed at evaluating methods that can realistically synthesize absent MRI modalities given multiple available images.
    The BraSyn-2023 dataset is based on the RSNA-ASNR-MICCAI BraTS 2021 dataset, comprising 1251 training cases, 219 validation cases, and 570 testing cases. The challenge required participants to handle scenarios where one of the four modalities was randomly absent (‘dropout’) in the test set provided for each subject, leaving only three modalities available. Participants’ algorithms were expected to generate plausible images for the missing modality. Figure 7 illustrates the BraSyn-2023 design, where one of the four modalities is randomly dropped during the validation and test phases, requiring participants to synthesize the missing modality and still achieve accurate segmentation. The synthesized images were evaluated on three criteria: their overall quality, the accuracy of segmentation within the generated images, and the effectiveness of a subsequent tumor segmentation algorithm when applied to the completed image set [28].
  7. Synthesis (Local)—inpainting: ASNR-MICCAI BraTS Local Synthesis of Tissue via Inpainting: In the BraTS 2023 inpainting challenge, participants were required to develop algorithmic solutions for synthesizing 3D healthy brain tissue in regions affected by glioma. This challenge arose from the need for improved tools to assist clinicians in decision-making and care provision, as most existing brain MR image analysis algorithms are optimized for healthy brains and may underperform on pathological images. These algorithms include, but are not limited to, brain anatomy parcellation, tissue segmentation, and brain extraction.
    The inpainting challenge addressed this issue by assigning the task of filling in pathological brain areas with synthesized healthy tissue, utilizing provided images and inpainting masks (See Figure 8). Since no actual healthy tissue data existed for tumor regions, surrogate inpainting masks created from the healthy portions of the brain were crafted using a specific protocol and provided to participants to train these algorithms. During training, participants were provided with T1 images featuring voided regions along with a corresponding void mask, the original T1 scans, and two additional masks: one for the tumor and one for the healthy area. This challenge exclusively utilized T1 scans for two main reasons: to reduce the computational demands on participants and to develop algorithms that could be generalized to other brain pathologies, as T1 scans are commonly included in MRI protocols for various conditions [29].
    In addition to enabling the application of standard brain image segmentation algorithms to tumor-affected brains, local inpainting also allows for the synthetic removal of tumor areas from images. This could deepen the understanding of the interplay between different brain regions and abnormal brain tissue, and is crucial for tasks such as brain tumor modeling. Moreover, inpainting helps manage local artifacts like B-field inhomogeneities, which sometimes impair the quality of brain tumor images.
  8. Evaluating Augmentations for BraTS: BraTS Challenge on Relevant Augmentation Techniques: The concept of data-centric machine learning focuses on enhancing model performance through the use of high-quality, meaningful training data. While data augmentation is recognized for boosting the robustness of machine learning models, the specific types of augmentations that benefit biomedical imaging remain uncertain. Participants were asked to develop methods to augment training data, aiming to enhance the robustness of a baseline model on newly introduced data.
    Software as a Medical Device (SaMD), as defined by the International Medical Device Regulators Forum (IMDRF), refers to software designed for medical use that operates independently of any hardware device. The challenge directly addressed concerns about algorithmic bias—where model predictions could be unfairly skewed against certain groups—and sought to improve algorithm robustness. Participants were tasked with creating computational methods that augmented a dataset of medical images, such that retraining a baseline AI/ML model on this augmented dataset led to improved accuracy and robustness on independent test data not previously exposed to the model. This approach would ensure that the AI/ML models used in SaMD are both effective and reliable, particularly in imaging-focused applications.
    The challenge used the RSNA-ASNR-MICCAI BraTS 2021 dataset to allow participants to develop and test their augmentation techniques. The goal was to improve a segmentation model with enhanced training data [10].
**Pre-Processing Data Protocol:** The standardized pre-processing protocol established by BraTS has been applied to all mpMRI scans.

**Figure 9:**
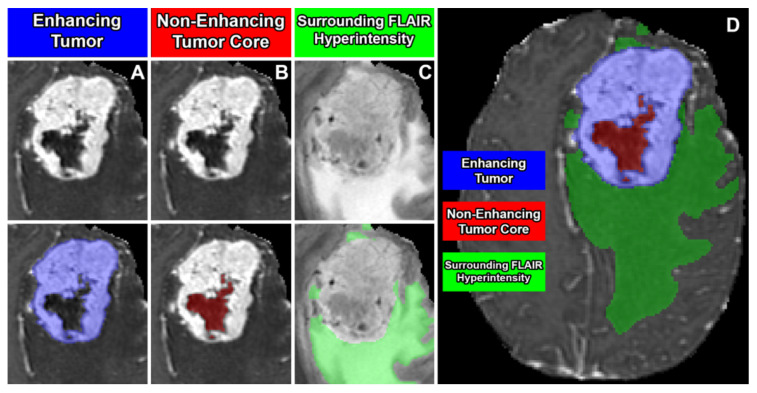
The image panels (**A**–**C**) identify the regions used for evaluating the performance of participating algorithms in some of the BraTS 2023 Challenge, including BraTS-Africa, BraTS-Meningioma, and BraTS-Metastasis. Specifically, panel (**A**) shows the enhancing tumor (ET, blue) in a T1Gd scan; panel (**B**) displays the non-enhancing tumor core (NETC, red) also in a T1Gd scan; and panel (**C**) highlights the surrounding non-enhancing FLAIR hyperintensity (SNFH, green) in a T2-FLAIR scan. Panel (**D**) illustrates the combined segmentations that generate the final tumor sub-region labels as introduced in the new segmentation labeling: enhancing tumor (ET, blue), non-enhancing tumor core (NETC, red), and edema (SNFH, green), as provided to the challenge participants. Figure adapted from [30], licensed under the non-exclusive license to distribute: https://arxiv.org/licenses/nonexclusive-distrib/1.0/license.html (accessed on 25 September 2024).

**Figure 10:**
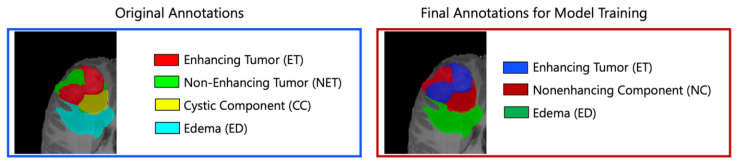
The (**left image**) displays the original tumor subregions: enhancing tumor (ET), non-enhancing tumor (NET), cystic component (CC), and edema (ED). The (**right image**) shows the final three-class labeling—enhancing tumor (ET), non-enhancing component (NC), and edema (ED)—provided to BraTS-PEDs 2023 participants. This consolidation ensures consistency with other BraTS 2023 challenges and facilitates comparative analyses across pediatric and adult glioma datasets. Figure adapted from [27], licensed under the CC BY 4.0: https://creativecommons.org/licenses/by/4.0/ (accessed on 25 September 2024).

**Annotation Method:**
  1. Segmentation—Adult Glioma Dataset: The BraTS Adult Glioma training and validation data were identical to the RSNA-ASNR-MICCAI BraTS 2021 dataset, and thus used the same annotation protocol. The only change, implemented starting in 2023, was a new naming convention where the ET label value was updated from ‘4’ to ‘3’. The annotations include the GD-enhancing tumor (ET—label 3), the peritumoral edematous/invaded tissue (ED—label 2), and the necrotic tumor core (NCR—label 1) [10].
  2. Segmentation—BraTS-Africa Dataset: All imaging data were reviewed and manually annotated by board-certified radiologists specializing in neuro-oncology, following the BraTS pre-processing and annotation protocols. Figure 9 demonstrates the new segmentation labeling introduced in 2023, which is also employed in the BraTS-Meningioma and BraTS-Metastasis 2023 challenge. This labeling system delineates three tumor sub-regions:
    (a) Enhancing tumor (ET): represents all tumor portions with a noticeable increase in T1 signal on post-contrast images compared to pre-contrast images, excluding adjacent blood vessels, intrinsic T1 hyperintensity, or abnormal signal in non-tumor tissues.
    (b) Non-enhancing tumor core (NETC): includes all non-enhancing tumor core areas, such as necrosis, cystic changes, calcification, and other non-enhancing components. Intrinsic T1 hyperintensity (e.g., intratumoral hemorrhage or fat) is also included.
    (c) Surrounding non-enhancing FLAIR hyperintensity (SNFH): covers the full extent of FLAIR signal abnormalities surrounding the tumor that are unrelated to the tumor core. For meningiomas, this corresponds to “vasogenic edema”, excluding non-tumor-related FLAIR abnormalities like prior infarcts or microvascular ischemic changes [10].
  3. Segmentation—meningioma: ASNR-MICCAI BraTS Intracranial Meningioma Challenge: Prior to manual segmentation, an automated pre-segmentation model using a deep convolutional neural network (nnU-Net) was used to produce initial multi-region segmentations. This model was initially trained on a dataset of 73 manually labeled studies from a single institution, all involving meningiomas that had undergone surgical resection. While effective, this initial training set introduced a bias that could reduce model performance for non-surgical cases. To mitigate this, the model was iteratively retrained during the challenge preparation phase using additional manually corrected cases from various sites, including non-surgical meningiomas. This iterative process aimed to enhance the model’s generalizability across diverse MRI appearances and reduce pre-segmentation bias.
    Manual corrections of the pre-segmented labels were performed by a diverse group of annotators, ranging from medical students to experienced neuroradiologists with over 10 years of expertise. Corrections were carried out using ITK-SNAP, an open-source tool for segmenting 3D and 4D biomedical images. Annotators were provided with detailed instructions, written descriptions of tumor sub-compartment compositions, and examples of common pre-segmentation errors to ensure consistency and reduce variability in corrections. After annotators completed their corrections, each case was reviewed by a trained neuroradiologist. Cases identified as incomplete or inaccurate were returned for additional refinement by a different annotator.
    For BraTS 2023 Meningioma, the task was to automatically segment intracranial meningiomas using a three-label system: enhancing tumor (ET), non-enhancing tumor core (NETC), and surrounding non-enhancing FLAIR hyperintensity (SNFH). For additional details on the pre-segmentation and annotation procedures, refer to the task manuscript: [30].
  4. Segmentation—brain metastases: ASNR-MICCAI BraTS Brain Metastasis Challenge: To ensure consistency in tumor labeling, a comprehensive annotation pipeline was established. This pipeline, designed to generate accurate ground truth (GT) labels, comprises five main stages: pre-segmentation, annotation refinement, technical quality control (QC), initial approval, and final approval. The pre-segmentation phase involved generating segmentation using three nnU-Net models trained on different datasets:
    (1) A model trained on the UCSF-BMSR MRI dataset to produce ET labels, fused with NETC and SNFH predictions from a model trained on the BraTS 2021 glioma dataset.
    (2) A model trained on the AURORA multicenter dataset to generate SNFH and tumor core (ET + NETC) labels.
    (3) A model trained on the Heidelberg dataset to segment SNFH and tumor core labels.
    The label fusion methods varied by label type: SNFH (label 2) was fused using the STAPLE algorithm to address systematic errors; ET (label 3) was combined using minority voting to improve the detection of small metastases; NETC (label 1) was derived solely from the UCSF-BMSR-trained model, overlapping ET and SNFH labels. Annotators were provided with pre-segmentations, fused segmentations, and subtraction images to refine annotations. Over 150 annotators, including students and neuroradiology experts, participated under coordinator supervision, receiving training through group reviews and lectures. Incomplete cases were returned for re-annotation. Additionally, quality control (QC) ensured proper alignment with the SRI24 atlas and validated the presence of all required segmentation masks. Each refined case underwent secondary review by another neuroradiologist, with discrepancies resolved directly by the reviewers. A final dataset review by an experienced neuroradiologist ensured uniformity and consistency. For more details, refer to the task manuscript [26].
  5. Segmentation—pediatric tumors: ASNR-MICCAI BraTS Pediatrics Tumor Challenge: Following pre-processing, the images were segmented into tumor subregions using two pediatric-specific automated deep-learning models. These models produced preliminary segmentations of four tumor subregions, as recommended by the Response Assessment in Pediatric Neuro-Oncology (RAPNO) working group for assessing treatment response in high-grade gliomas and DIPGs. The subregions included the following:
    - Enhancing tumor (ET): Bright areas on T1 post-contrast images compared to T1 pre-contrast, with mild enhancement verified by comparing normal brain signal intensity.
    - Cystic component (CC): Very bright on T2 and dark on T1CE, resembling cerebrospinal fluid (CSF) and located within the tumor, either centrally or peripherally.
    - Non-enhancing tumor (NET): Any abnormal signal within the tumor not classified as enhancing or cystic.
    - Peritumoral edema (ED): Bright, finger-like areas on FLAIR scans, preserving underlying brain structure and surrounding the tumor.
    The automated segmentations served as the basis for manual refinement by volunteer neuroradiology experts using ITK-SNAP software. Annotators were provided with all four mpMRI sequences and the fused segmentation volume to guide their corrections. Refined segmentations were then reviewed by three board-certified neuroradiologists. Cases were either approved or returned for additional adjustments in an iterative process until the segmentations were deemed acceptable.
    For consistency with other BraTS 2023 challenges and to facilitate integration with adult glioma datasets, participants were provided with three final segmentation labels, enhancing tumor (ET), peritumoral edematous area (ED), and non-enhancing component (NC), which is a combination of non-enhancing tumor, cystic component, and necrosis [27]. Figure 10 illustrates this consolidation process: the left image shows the initial four-class subregions (ET, NET, CC, ED), while the right image depicts how these were merged into the final three-class labeling scheme used for model training.
  6. Synthesis (Global)—missing MRI: ASNR-MICCAI BraTS MRI Synthesis Challenge (BraSyn): The BraSyn-2023 dataset is based on the RSNA-ASNR-MICCAI BraTS 2021 dataset and, therefore, shares the same annotation protocol [28].
  7. Synthesis (Local)—inpainting: ASNR-MICCAI BraTS Local Synthesis of Tissue via Inpainting: For the BraTS 2023 challenge, T1 sequence images were used to generate two types of masks for participants. The first mask type delineates tumor-affected areas, while the second identifies likely healthy brain regions. Healthy brain masks were created by sampling existing tumor shapes and placing them in regions distant from tumors, enabling realistic training and evaluation of inpainting methods.
    The procedure for generating inpainting masks involves several steps. A pool of 1429 tumor masks was extracted from 1251 brains in the BraTS 2023 Adult Glioma dataset, selecting disconnected tumor compartments with at least 800 voxels. Masks were chosen based on tumor size, with small masks assigned to large tumors and vice versa, ensuring variability. Masks underwent transformations, including mirroring and random rotations, and were placed at semi-random positions distant from the tumor. Validity checks ensured masks met specific criteria, such as maintaining a minimum distance of five voxels from the tumor and limited overlap with the background. Once valid, both healthy and tumor masks were provided to participants. Participants were encouraged to enhance the process and create additional masks for training. To ensure healthy masks were accurate, medical experts manually reviewed and selected the best candidates from algorithm-generated options. Larger masks were preferred where possible to counteract a statistical bias toward smaller ones. For more details, refer to the task manuscript [29].
  8. Evaluating Augmentations for BraTS: BraTS Challenge on Relevant Augmentation Techniques: The dataset is based on the RSNA-ASNR-MICCAI BraTS 2021 dataset and, therefore, shares the same annotation protocol.

**Multi-Center Imaging Data Acquisition Details:**
For each task, a detailed list of contributors and their affiliations is available in the respective manuscripts or in the complete contributor list here: [10].
  1. Segmentation—adult glioma: RSNA-ASNR-MICCAI BraTS Continuous Evaluation Challenge: Same as BraTS 2021.
  2. Segmentation—BraTS-Africa: BraTS Challenge on Sub-Sahara-Africa Adult Glioma: Contributions to the BraTS-Africa data came from a total of four medical professionals across several prestigious institutions in Nigeria.
  3. Segmentation—meningioma: ASNR-MICCAI BraTS Intracranial Meningioma Challenge: The dataset for the ASNR-MICCAI BraTS Intracranial Meningioma Challenge comprises contributions from six institutions, all located in the United States (USA).
  4. Segmentation—brain metastases: ASNR-MICCAI BraTS Brain Metastasis Challenge: The BraTS-METS dataset included mpMRI scans from diverse institutions, representing the variability in imaging protocols and equipment reflective of global clinical practices. Eleven source institutions contributed to the Brain Metastases Dataset, with nine located in the United States, one in Germany, and one in Egypt.
  5. Segmentation—pediatric tumors: ASNR-MICCAI BraTS Pediatrics Tumor Challenge: Eight source institutions contributed to the dataset, all located in the United States (USA).
  6. Synthesis (Global)—missing MRI: ASNR-MICCAI BraTS MRI Synthesis Challenge (BraSyn): The BraSyn-2023 dataset is based on the RSNA-ASNR-MICCAI BraTS 2021 dataset. Therefore, data contributors are the same as BraTS 2021.
  7. Synthesis (Local)—inpainting: ASNR-MICCAI BraTS Local Synthesis of Tissue via Inpainting: BraTS Local Inpainting Challenge exclusively employs T1 scans from the multi-modal BraTS 2023 glioma segmentation challenge; therefore, the data contributors are the same as those for BraTS 2021.
  8. Evaluating Augmentations for BraTS: BraTS Challenge on Relevant Augmentation Techniques: The dataset is based on the RSNA-ASNR-MICCAI BraTS 2021 dataset. Therefore, data contributors are the same as BraTS 2021.

### 4.11. Dataset Review: BraTS Challenge 2024

**Number of tasks:** 10:
  1. Segmentation—adult glioma post-treatment: BraTS Adult Glioma Post Treatment Challenge;
  2. Segmentation—BraTS-Africa: BraTS Challenge on Sub-Sahara-Africa Adult Glioma;
  3. Segmentation—meningioma radiotherapy: BraTS Meningioma Radiotherapy Challenge;
  4. Segmentation—brain metastases: ASNR-MICCAI BraTS Brain Metastasis Challenge;
  5. Segmentation—pediatric tumors: ASNR-MICCAI BraTS Pediatrics Tumor Challenge;
  6. Segmentation—generalizability: ASNR-MICCAI BraTS Generalizability Across Brain Tumors;
  7. Synthesis (Global)—missing MRI: ASNR-MICCAI BraTS MRI Synthesis Challenge (BraSyn);
  8. Synthesis (Local)—inpainting: ASNR-MICCAI BraTS Local Synthesis of Tissue via Inpainting;
  9. Evaluating Augmentations for BraTS: BraTS Challenge on Relevant Augmentation Techniques;
  10. Pathology.

**Number of classes for tasks 1:** four class labels:
  - Label 1—NETC: non-enhancing tumor core;
  - Label 2—SNFH: surrounding non-enhancing FLAIR hyperintensity;
  - Label 3—ET: enhancing tumor;
  - Label 4—RC: resection cavity;
  - Label 0: everything else.

**Number of classes for task 3:** one class label:
  - Label 1—GTV: gross tumor volume.

**Number of classes for tasks 9:** three class labels:
  - Label 1—NCR: necrotic tumor core;
  - Label 2—ED: peritumoral edematous/invaded tissue;
  - Label 3—ET: GD-enhancing tumor;
  - Label 0: everything else.

**Number of classes for tasks 2–4:** three class labels:
  - Label 1—NETC: non-enhancing tumor core;
  - Label 2—SNFH: surrounding non-enhancing FLAIR hyperintensity;
  - Label 3—ET: enhancing tumor;
  - Label 0: everything else.

**Number of classes for tasks 5:** four class labels:
  - Label 1—ET: enhancing tumor;
  - Label 2—NET: non-enhancing tumor;
  - Label 3—CC: cystic component;
  - Label 4—ED: peritumoral edema;
  - Label 0: everything else.

**Number of classes for tasks 10:** nine class labels:
  - Label 1—CT: presence of cellular tumor;
  - Label 2—PN: pseudopalisading necrosis;
  - Label 3—MP: areas abundant in microvascular proliferation;
  - Label 4—NC: geographic necrosis;
  - Label 5—IC: infiltration into the cortex;
  - Label 6—WM: penetration into white matter;
  - Label 7—LI: leptomeningeal infiltration;
  - Label 8—DM: regions dense with macrophages;
  - Label 9—PL: presence of lymphocytes;
  - Label 0: everything else.

**MRI modalities:** All four modalities were available: T1-weighted, native image (T1); T1-weighted, contrast-enhanced (Gadolinium) image (T1c); T2-weighted image (T2); and T2-weighted FLAIR image (FLAIR).
Primarily due to computational constraints, the synthesis (local)—inpainting task exclusively utilized T1 scans. In addition, the 2024 Meningioma Radiotherapy Challenge dataset utilized only T1-weighted scans in native acquisition space. This approach is designed to mimic the data typically available for most radiotherapy planning scenarios.

**Challenge Data:**
  1. Segmentation—adult glioma post-treatment: BraTS Adult Glioma Post Treatment Challenge: Gliomas are the most common malignant primary brain tumors in adults, with diffuse gliomas being particularly prevalent. Treatment typically involves a combination of surgery, radiation, and systemic therapies tailored to the tumor’s characteristics and the patient’s condition. MRI remains the gold standard for monitoring diffuse gliomas post-treatment, offering critical insights into tumor size, location, and morphological changes, which are essential for guiding treatment adjustments and predicting clinical outcomes.
    The 2024 BraTS challenge introduced a focus on post-treatment gliomas for the first time. Task 1 aimed to develop an automated brain tumor segmentation algorithm for high- and low-grade diffuse gliomas in post-treatment MRI, including a new subregion: the resection cavity (RC). Data and algorithms generated from this challenge have the potential to aid in objectively assessing residual tumor volume, improving treatment planning and outcome prediction [11].
  2. Segmentation—BraTS-Africa: BraTS Challenge on Sub-Sahara-Africa Adult Glioma: The BraTS-Africa Challenge, first launched in 2023, introduced labeled datasets specifically for adult populations in Sub-Saharan Africa. Building on its success, the second BraTS-Africa Challenge (BraTS-Africa 2024) was organized. The MICCAI-CAMERA-Lacuna Fund BraTS-Africa 2024 Challenge offered the largest publicly available annotated retrospective dataset of pre-operative glioma cases in adult Africans, including both low-grade glioma (LGG) and glioblastoma/high-grade glioma (GBM/HGG). Participants were tasked with developing segmentation methods using the clinically acquired BraTS-Africa training data, supplemented with training and validation datasets from the BraTS 2023 Adult Glioma Challenge [11].
  3. Segmentation—meningioma radiotherapy: BraTS Meningioma Radiotherapy Challenge: Meningioma is the most common primary intracranial tumor, with about 80% being benign and effectively managed with positive outcomes. However, higher-grade meningiomas (WHO grades 2 and 3) pose greater health risks and have higher recurrence rates. They are primarily treated with radiation therapy, which may be used as the main treatment, as a postoperative adjunct, or for recurring cases. Accurate segmentation of the gross tumor volume (GTV) is critical for radiotherapy planning but remains complex and time-intensive, with no reliable automated solutions currently available.
    The BraTS 2024 Meningioma Radiotherapy Segmentation Challenge aimed to bridge existing gaps by developing algorithms capable of automatically segmenting gross tumor volumes (GTVs) in cranial and facial meningiomas from radiotherapy planning MRI scans.
    The BraTS-MEN-RT challenge exclusively includes radiotherapy planning brain MRI scans, either preoperative or postoperative, with tumors that are radiographically or pathologically consistent with meningioma. These scans consist of a single series (T1-weighted imaging) in native acquisition space, reflecting typical radiotherapy planning scenarios. This approach replaces the multi-sequence, co-registered MRI scans used in each of the BraTS 2023 automated segmentation challenges [11].
  4. Segmentation—brain metastases: ASNR-MICCAI BraTS Brain Metastasis Challenge: The challenge focused on developing versatile autosegmentation algorithms capable of precisely delineating brain metastases of varying sizes. These algorithms aim to streamline monitoring and management in both pre- and post-treatment settings, offering the potential to significantly enhance patient care and treatment planning [11].
  5. Segmentation—pediatric tumors: ASNR-MICCAI BraTS Pediatrics Tumor Challenge: Building on the first BraTS-PEDs Challenge in 2023, the 2024 BraTS-PEDs Challenge expanded to include a larger and more diverse dataset from additional institutions, enhancing its scope and clinical relevance.
    The 2024 challenge provided the largest publicly available annotated cohort of high-grade pediatric gliomas, including astrocytoma and diffuse midline glioma (DMG)/diffuse intrinsic pontine glioma (DIPG).
    This year, the challenge introduced significant updates to the processing pipeline and tumor subregion evaluation. The evaluated regions now include: (i) enhancing tumor (ET); (ii) non-enhancing tumor (NET); (iii) cystic component (CC); (iv) edema (ED); (v) tumor core (TC), which combines ET, NET, and CC; and (vi) the whole tumor (WT) [31].
  6. Segmentation—generalizability: ASNR-MICCAI BraTS Generalizability Across Brain Tumors: While segmentation is a widely studied medical image processing task, the BraTS Generalizability Across Tumors (BraTS-GoAT) Challenge emphasizes algorithmic generalizability across various tumor types and patient populations. The goal was to develop a segmentation algorithm that can adapt to different brain tumor types with limited training data.
    Candidate algorithms should demonstrate the ability to generalize across: lesion types (variability in lesion count, size, and brain location), institutions (differences in MRI scanners and acquisition protocols), and demographics (patient age, sex, and other factors). Although the segmentation mask labels remained consistent (“1” for NCR (necrosis), “2” for ED (edema/invaded tissue), “3” for ET (enhancing tumor), and “0” for everything else), the prevalence of each label varied within and across tumor types, reflecting the diverse imaging characteristics of the lesions.
    The challenge used preoperative MRI data from BraTS 2023 tasks (1 through 5), focusing on assessing the ability of algorithms to generalize beyond individual datasets and across multiple clinical applications [11].
  7. Synthesis (Global)—missing MRI: ASNR-MICCAI BraTS MRI Synthesis Challenge (BraSyn): Continuing from the success of the BraSyn 2023 Challenge, the 2024 iteration maintained the goal of evaluating image synthesis methods that create realistic image contrasts from available MRI modalities to aid automated brain tumor segmentation. The BraSyn-2024 dataset, identical in task to BraSyn-2023 [11].
  8. Synthesis (Local)—inpainting: ASNR-MICCAI BraTS Local Synthesis of Tissue via Inpainting: The ASNR-MICCAI BraTS Local Synthesis of Tissue via Inpainting Challenge continued for BraTS 2024 with the same design, task, evaluation metrics, and ranking scheme as the previous year. This iteration introduced an additional test set of 277 patients from the BraTS Meningioma Challenge, enabling an assessment of how well algorithms generalize to other pathologies and imaging data acquired from different MRI scanners [29].
  9. Evaluating Augmentations for BraTS: BraTS Challenge on Relevant Augmentation Techniques: BraTS 2024 Challenge on Relevant Augmentation Techniques shared the same objectives and dataset as the BraTS 2023 Challenge on Relevant Augmentation Techniques.
  10. Pathology: glioblastoma has a poor prognosis, with median survival ranging from 12 to 18 months with treatment and only 4 months without. Its infiltrative nature and heterogeneous molecular and micro-environmental profiles make treatment challenging. Accurate diagnosis and assessment of tumor heterogeneity are critical for selecting effective therapies and potentially improving patient outcomes. The BraTS-Path Challenge leverages whole slide histopathology images (WSI) to enhance the understanding of glioblastoma by detecting various morpho-pathological features in digitized tissue sections. It draws on the gold-standard histopathology-based approach, which traditionally focuses on identifying features such as cellular tumor regions, necrosis, microvascular proliferation, cortical infiltration, and immune cell presence. By providing a robust dataset, the challenge aims to develop deep-learning models capable of automatically classifying these distinct tumor sub-regions with varied histological profiles. These models are designed to mimic and extend the gold-standard process, supporting more consistent diagnosis and grading, thus enhancing both research and clinical applications [32].
    The dataset included a retrospective, multi-institutional cohort of de novo diffuse gliomas. Expert neuropathologists annotated and segmented histological regions into patches for classification based on specific characteristics. The dataset consists of 195,000 training data points from 130 digitized tissue sections, 25,000 validation data points from 18 digitized tissue sections, and 60,000 testing data points from 40 digitized tissue sections. Nine histologic areas of interest are considered as classes [11].

**Pre-Processing Data Protocol:** The standardized pre-processing protocol established by BraTS has been applied to all datasets except the following, which exhibit some slight differences:
  - Task 3 Segmentation—meningioma radiotherapy: All images underwent standardized preprocessing, including conversion from DICOM and DICOM-RT to NIfTI format using dcmrtstruct2nii, followed by automated defacing with the AFNI toolbox. Cases where the AFNI defacing algorithm removed the meningioma entirely from the field of view (e.g., anterior intraorbital meningiomas) were excluded from the dataset [33].
  - Task 5 Segmentation—pediatric tumors: ASNR-MICCAI BraTS Pediatrics Tumor Challenge: All mpMRI scans were processed through a three-step pipeline. First, pre-processing was carried out using the “BraTS Pipeline”, a standardized approach that is publicly available through the Cancer Imaging Phenomics Toolkit (CaPTk) and Federated Tumor Segmentation (FeTS) tool. Next, a pediatric-specific automated defacing method was applied to ensure patient anonymity. Finally, tumor subregion segmentation was completed using a pediatric autosegmentation method.
    It is important to note that the BraTS-PEDs 2024 dataset does not include skull stripping.
  - Task 10—pathology: The preprocessing pipeline for H&E-stained FFPE tissue sections of glioblastoma multiform (GBM) was specifically designed to facilitate detailed histological analysis.
    (1) Patch Extraction and Standardization: Tissue sections annotated by expert neuropathologists are segmented into standardized 512 × 512-pixel patches. This size minimizes boundary noise and ensures comprehensive coverage of annotated regions.
    (2) Quality Control: Only high-quality sections are included, excluding those with artifacts such as tissue folding, pen markings, or glass slippage.
    (3) Patch Classification: Each patch is categorized based on its histologic features, which are used throughout training, validation, and testing to evaluate the challenges and accuracy of feature detection.
    This structured pipeline ensures high-quality data for histological studies, with each patch treated as an independent unit for comprehensive analysis [32].

**Figure 11:**
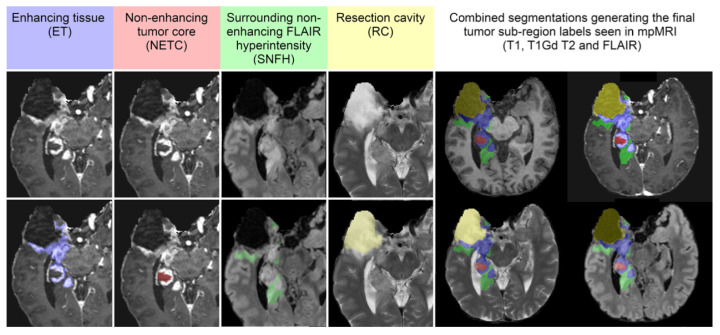
Sub-regions considered in the 2024 BraTS post-treatment glioma challenge include the enhancing tissue (blue), the non-enhancing tumor core (red), the surrounding non-enhancing FLAIR hyperintensity (green), and the newly introduced label for the resection cavity (yellow). Figure adapted from [34], licensed under the CC BY 4.0: https://creativecommons.org/licenses/by/4.0/ (accessed 15 October 2024).

**Figure 12:**
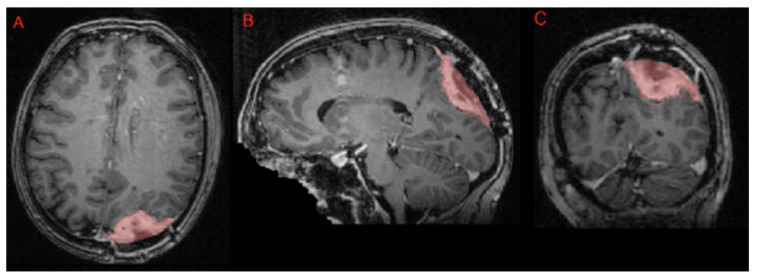
Brain MRI of a patient showing a postoperative left parietal meningioma target volume (red), as delineated by the treating institution, presented in different planes: Axial view in panel (**A**), sagittal view in panel (**B**), and coronal view in panel (**C**). Figure adapted and modified from: [33], licensed under the CC BY 4.0: https://creativecommons.org/licenses/by/4.0/ (accessed 12 October 2024).

**Figure 13:**
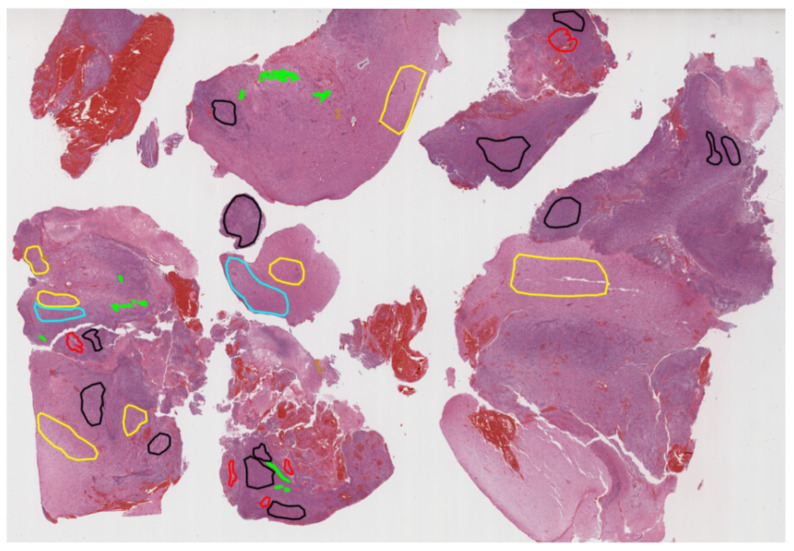
Example of a whole slide histopathology image (WSI) from the BraTS-Path 2024 Challenge, illustrating glioblastoma (GBM) pathology sub-regions considered for evaluation. The image includes pseudopalasading necrosis (Red), microvascular proliferation (Green), necrosis (Blue), infiltration into the cortex (Yellow), cellular tumor (Black), and penetration into white matter (Sky blue). Additional histological features are not present in this particular slide but are annotated in other WSIs within the dataset. Figure adapted from [32], licensed under the CC BY 4.0: https://creativecommons.org/licenses/by/4.0/ (accessed 15 October 2024).

**Annotation Method:**
  1. Segmentation—adult glioma post-treatment: BraTS Adult Glioma Post Treatment Challenge: The BraTS 2024 Post-Treatment Adult Glioma Challenge builds upon the framework established in the BraTS 2021–2023 challenges by introducing modifications tailored specifically to the post-treatment context. All imaging datasets were manually annotated by one to four raters using a standardized, clinically approved protocol developed by expert neuroradiologists and radiation oncologists. Annotators followed detailed guidelines, including examples of challenging cases, to ensure consistency. For a full description of the protocol, refer to the challenge manuscript: [34].
    Preprocessed MRI volumes were segmented using five nnU-Net-based pre-segmentation methods. These outputs were fused using the STAPLE algorithm to generate consensus segmentations. Additionally, digital subtraction images (T1-Gd minus T1) were provided to radiologists to improve annotation accuracy.
    As shown in Figure 11, the annotations included the following tumor subregions: the enhancing tissue (ET—label 3); the surrounding non-enhancing FLAIR hyperintensity (SNFH—label 2); the non-enhancing tumor core (NETC—label 1), and the newly introduced resection cavity (RC—label 4). This expanded labeling scheme highlights the complexities of post-treatment imaging—such as surgical cavities and residual tumors—thereby enabling researchers to investigate regrowth patterns, therapy response, and other clinically relevant factors in greater detail. Annotations were iteratively refined and reviewed by board-certified neuroradiologists. This process continued until segmentations met the required quality standard for public release. Test data annotations underwent review by two sets of annotators and approvers to ensure inter-rater reliability [11,34].
  2. Segmentation—BraTS-Africa: BraTS Challenge on Sub-Sahara-Africa Adult Glioma: Same annotation protocol as BraTS-Africa Challenge 2023.
  3. Segmentation—meningioma radiotherapy: BraTS Meningioma Radiotherapy Challenge: For the BraTS-MEN-RT 2024 challenge, a single “target volume” label was employed, representing both the gross tumor volume (GTV) and any at-risk postoperative site. Consequently, the definition of GTV varies depending on the radiotherapy planning context:
    - Preoperative setting: The target volume includes the portion of the tumor visible on T1c brain MRI.
    - Postoperative setting: The target volume includes the resection bed and any residual enhancing tumor (ET) visible on T1c brain MRI.
    Figure 12 provides an example of postoperative meningioma data with this target volume label, illustrating how the resection bed and any residual enhancing tumor are encompassed within the single “target volume” annotation.
    For cases without gross tumor volume (GTV) labels provided by the treating institution (about 10% of the dataset), an automated pre-segmentation algorithm using nnU-Net was applied. All cases, whether pre-segmented or labeled by the institution, underwent manual review and correction by a senior radiation oncology resident. Subsequently, a fellowship-trained neuroradiologist (“approver”) performed a final review to ensure data quality. Manual reviews and corrections were conducted using ITKSnap [33].
  4. Segmentation—brain metastases: ASNR-MICCAI BraTS Brain Metastasis Challenge: The dataset includes retrospective treatment-naive brain metastasis MRI scans, adhering to the BraTS 2023 annotation protocol.
    The post-treatment brain metastasis MRI scans, sourced from an external dataset, are expected to follow a five-label system that includes the necrotic core of tumor (NCR), FLAIR hyperintensity (SNFH), enhancing tumor (ET), hemorrhage (HM), and resection cavity (RC). While the detailed annotation protocol for the post-treatment cases has not yet been released, additional information will be provided in the forthcoming challenge manuscript, with updates available on the official challenge website (Synapse.org) [11].
  5. Segmentation—pediatric tumors: ASNR-MICCAI BraTS Pediatrics Tumor Challenge: Following pre-processing and defacing, a pediatric automated segmentation method was employed to preliminarily segment tumors into four main subregions: enhancing tumor (ET; label 1), non-enhancing tumor (NET; label 2), cystic component (CC; label 3), and peritumoral edema (ED; label 4).
    The automated segmentation outputs were manually revised by volunteer neuroradiology experts with varying levels of experience, following established annotation guidelines. Refinements were conducted using ITK-SNAP software. Afterward, the refined segmentations were reviewed by three board-certified neuroradiologists. Cases deemed incomplete or inaccurate were returned to the annotators for further adjustments. This iterative review process continued until the segmentations were approved by the neuroradiologists [31]. Note that for the BraTS-PEDs 2024 challenge, the tumor sub-regions considered for evaluation returned to the original four, as initially planned for the BraTS-PEDs 2023 challenge but not implemented that year (see left image in Figure 10).
  6. Segmentation—generalizability: ASNR-MICCAI BraTS Generalizability Across Brain Tumors: In BraTS-GoAT, preoperative MRI data from the BraTS 2023 challenge is utilized, specifically from tasks 1, 2, 3, 4, and 5. The sub-regions considered for evaluation are the enhancing tumor (ET), the tumor core (TC), and the whole tumor (WT). The provided segmentation labels have values of: “1” for NCR (necrosis), “2” for ED (edema/invaded tissue), “3” for ET (enhancing tumor), and “0” for everything else [11]. Details regarding the annotation protocol have not yet been disclosed.
  7. Synthesis (Global)—missing MRI: ASNR-MICCAI BraTS MRI Synthesis Challenge (BraSyn): Same as BraSyn 2023 Challenge.
  8. Synthesis (Local)—inpainting: ASNR-MICCAI BraTS Local Synthesis of Tissue via Inpainting: Same as BraTS Local Inpainting 2023 Challenge.
  9. Evaluating Augmentations for BraTS: BraTS Challenge on Relevant Augmentation Techniques: Same as BraTS Challenge on Relevant Augmentation 2023.
  10. Pathology: The annotation process adhered to a clinically approved protocol, defined by expert neuropathologists, which provided detailed instructions on segmenting each histologic feature. For further details, refer to the challenge manuscript: [32]. Each case was assigned to an annotator–approver pair. To assess inter-rater variability, three cases were annotated by all annotators. Annotators varied in experience and rank, while approvers were highly experienced, board-certified neuropathologists with over 10 years of expertise.
    Once annotations were completed, they were reviewed by the approvers, who evaluated their quality alongside the corresponding tissue sections. If the initial annotations on a tissue section produced fewer than approximately 1500 patches, that section was returned to the annotators for further refinement. This iterative process continued until the approvers approved the annotations. The segmented regions were then divided into patches and classified based on specific histologic features. This established a classification task focused on accurately identifying patches with distinct morphological characteristics [32].
    Histologic areas of interest for classification included the following:
    (1) Presence of cellular tumor (CT);
    (2) Pseudopalisading necrosis (PN);
    (3) Areas abundant in microvascular proliferation (MP);
    (4) Geographic necrosis (NC);
    (5) Infiltration into the cortex (IC);
    (6) Penetration into white matter (WM);
    (7) Leptomeningeal infiltration (LI);
    (8) Regions dense with macrophages (DMs);
    (9) Presence of lymphocytes (PLs).
    An example of these annotated histological sub-regions is illustrated in Figure 13, which displays a whole slide histopathology image (WSI) from the BraTS-Path 2024 Challenge. The figure delineates various glioblastoma (GBM) sub-regions with distinct colors, capturing critical pathological features. These comprehensive annotations are essential for training machine learning models, facilitating more precise and automated histopathological analysis of brain tumors and improving diagnostic accuracy in neuro-oncology.

**Multi-Center Imaging Data Acquisition Details:** For each task, a detailed list of contributors and their affiliations is available in the respective manuscripts or in the complete contributor list here: [11].
  1. Segmentation—adult glioma post-treatment: BraTS Adult Glioma Post Treatment Challenge: BraTS mpMRI scans come from multiple contributing institutions using various scanners and clinical protocols. Data from seven institutions contributed to the dataset, with locations across the United States and one in Germany.
  2. Segmentation—BraTS-Africa: BraTS Challenge on Sub-Sahara-Africa Adult Glioma: The BraTS-Africa 2024 dataset saw an expansion in the number of contributors compared to the 2023 edition, reflecting a substantial enlargement of the dataset. This increase includes new collaborations from additional healthcare institutions across several African countries, including Tanzania and Ghana.
  3. Segmentation—meningioma radiotherapy: BraTS Meningioma Radiotherapy Challenge: 750 radiotherapy planning T1c brain MRI scans were contributed from seven academic medical centers across the United States and the United Kingdom.
  4. Segmentation—brain metastases: ASNR-MICCAI BraTS Brain Metastasis Challenge: Contributions to the dataset came from ten institutions located across the United States, Germany, and Egypt.
  5. Segmentation—pediatric tumors: ASNR-MICCAI BraTS Pediatrics Tumor Challenge: The image acquisition protocols and MRI equipment vary between institutions, leading to differences in image quality within the provided cohort. The dataset from the CBTN-CONNECT-DIPGr-ASNR-MICCAI BraTS-PEDs initiative includes contributions from a total of eight institutions, all located in the United States.
  6. Segmentation—generalizability: ASNR-MICCAI BraTS Generalizability Across Brain Tumors: The data contributors include those involved in challenges 1, 2, 3, 4, and 5 from the 2023 edition. This collaborative effort provides a comprehensive and diverse dataset, supporting the development of models with improved generalizability across various brain tumor types.
  7. Synthesis (Global)—missing MRI: ASNR-MICCAI BraTS MRI Synthesis Challenge (BraSyn): Same as BraTS 2021.
  8. Synthesis (Local)—inpainting: ASNR-MICCAI BraTS Local Synthesis of Tissue via Inpainting: Same as BraTS 2021.
  9. Evaluating Augmentations for BraTS: BraTS Challenge on Relevant Augmentation Techniques: Same as BraTS 2021.
  10. Pathology: Contributions to the dataset came from ten institutions across the United States and one in Italy.
  Table 3 provides a concise summary of each challenge’s main characteristics—such as the tasks, MRI modalities employed, number of classes, contributing institutions, and whether it includes data from low- and middle-income countries (LMICs).

### 4.12. BraTS Challenge 2025 Overview

The BraTS Challenge 2025 is currently in planning, and new information about its expanded scope is now available. In collaboration with leading clinical organizations such as AI-RANO, RSNA, ASNR, NIH, FDA, ASFNR, and CBTN, the BraTS 2025 Lighthouse Challenge will extend its focus to address additional clinical needs. These include the longitudinal assessment of brain tumor response, the generalizability of tumor segmentation methods across different entities, and the inclusion of tumor types for which there is currently limited annotated data. As a novelty, algorithmic performance in 2025 will also be benchmarked against human expert inter-rater variability, further elucidating the clinical applicability of BraTS solutions.

The challenge will release 12 unique tasks addressing critical topics, including the following:

- Pre- and Post-Treatment Adult Glioma;
- Preoperative Meningioma Tumor Segmentation;
- Pre-Radiotherapy Intracranial Meningioma;
- Pre- and Post-Treatment Brain Metastases;
- Brain Glioma in the Underserved Sub-Saharan African Patient Population;
- Pre-Treatment Pediatric Tumor Patients in Partnership with Multiple Related Societies;
- Generalizability of Segmentation Methods Across Tumors;
- MRI Global Synthesis;
- MRI Local Inpainting;
- Assessing the Heterogeneous Histologic Landscape of Glioma;
- Predicting the Tumor Response During Therapy.

Notably, Task 1 introduces the first dataset that combines pre- and post-treatment glioma data. The purpose of this subchallenge is to develop an automated multi-compartment segmentation algorithm for high- and low-grade diffuse gliomas on MRI. Data and algorithms from this task will enable the creation of tools for objective tumor volume assessment, aiding treatment planning and outcome prediction.

Additionally, the BraTS 2025 Brain Metastases dataset comprises a retrospective collection of pre- and post-treatment brain metastases mpMRI scans obtained from multiple institutions under standard clinical conditions. This diverse data, gathered from various equipment and imaging protocols, reflects the broad spectrum of image quality observed in real-world clinical practices.

Furthermore, the BraTS 2025 Meningioma Radiotherapy Segmentation Challenge will introduce a significant innovation compared to its 2024 predecessor: the inclusion of multiple annotations from different annotators on a subset of the testing cohort. This modification allows participant models to be evaluated against a range of human annotations, facilitating an assessment of intra- and inter-observer variability in meningioma radiotherapy segmentation.

This overview summarizes the new elements that BraTS 2025 is set to introduce and provides a glimpse into the future directions of the challenge [35].

## 5. BraTS Challenge Results over the Years: SOTA Development

Building on the rich foundation laid by the extensive and increasingly complex BraTS datasets detailed previously, we now turn our focus to the evolution of segmentation algorithms that have been developed through the annual BraTS challenges from 2012 to 2024. Each year has seen the introduction of state-of-the-art (SOTA) technologies, meticulously crafted to meet and leverage the complexities introduced by the expanding and diversifying datasets. This section reviews these yearly technological breakthroughs, tracing how advancements in machine learning have been tightly coupled with enhancements in dataset detail and diversity. Through this chronological exploration, we highlight the pivotal role of the BraTS challenges in driving forward the precision and capabilities of brain tumor segmentation models, illustrating the direct impact of evolving data on algorithmic innovation:

**2012:** The BraTS challenge started with models achieving Dice scores between 0.14 and 0.70 for whole tumors (WT) and 0.09–0.37 for tumor core (TC). The models were mainly based on Random Forests, logistic regression, and Markov Random Fields. The best-performing model was a Random Forest-based approach, achieving a Dice score of 0.70 and 0.25 for the whole tumor and tumor core, respectively.
**2013:** Dice scores improved, ranging from 0.71 to 0.87 for whole tumors (WT), from 0.46 to 0.78 for tumor core (TC), and from 0.52 to 0.74 for enhancing tumor (ET). This year saw a continuation of the success of RF-based methods that utilized advanced texture features and multimodal MRI data.
**2014:** Introduced deep convolutional networks alongside traditional RF methods, marking the beginning of deep learning’s influence in medical image segmentation. U-Net, a CNN architecture, began to emerge as a central model, with top models reaching Dice scores up to 0.87 for whole tumors.
**2015:** CNNs dominated, driven by their high segmentation accuracy. The InputCascadeCNN, a sophisticated 2D CNN architecture, performed well on the BraTS 2013 dataset with Dice scores of 0.88, 0.79, and 0.73 for whole tumor (WT), tumor core (TC), and enhancing tumor (ET), respectively. Architectural enhancements included residual connections and densely connected layers.
**2016:** DeepMedic, an 11-layer 3D CNN with dual pathways and residual connections, led the challenge, achieving Dice scores of 0.89, 0.76, and 0.72 for whole tumor (WT), tumor core (TC), and enhancing tumor (ET), respectively. This solidified the position of 3D CNNs in volumetric segmentation tasks.
**2017:** Over 50 papers were submitted, predominantly leveraging U-Net and DeepMedic architectures. Ensemble methods, like EMMA (Ensemble of Multiple Models and Architectures), became prominent. EMMA, comprising two different DeepMedics, two fully convolutional networks, and a U-Net, won with Dice scores of 0.90, 0.82, and 0.75 for whole tumor (WT), tumor core (TC), and enhancing tumor (ET), respectively, on blinded validation.
**2018:** The challenge witnessed a dominant use of CNNs, particularly U-net architectures and their ensembles, which formed the basis of the top submissions. A notable trend was the integration of advanced CNN architectures like ResNet and DenseNet. The top-ranked model employed an asymmetric encoder–decoder architecture with a larger encoder to extract more features, complemented by a variational auto-encoder branch for input reconstruction. This innovative approach, alongside an ensemble of 10 individually trained models, led to impressive Dice scores of 0.91 for whole tumor (WT), 0.86 for tumor core (TC), and 0.82 for enhancing tumor (ET). The methods employed underscored a shift towards more sophisticated, ensemble-based strategies [16].
**2019:** The Two-Stage Cascaded U-Net introduced a coarse-to-fine segmentation approach, achieving Dice scores of 0.88796 for whole tumors (WT), 0.83697 for tumor core (TC), and 0.83267 for enhancing tumor (ET). This model effectively balanced precision across tumor subregions [36].
**2020:** The nnU-Net framework dominated, employing BraTS-specific optimizations such as batch Dice loss, aggressive data augmentation, and post-processing tailored to handle small enhancing tumor (ET) regions. Dice scores on the test dataset were 0.8895 for whole tumors (WT), 0.8506 for tumor core (TC), and 0.8203 for enhancing tumors (ET). The ensemble approach balanced performance across tumor subregions [37].
**2021:** The winning model extended nnU-Net, incorporating a larger network architecture, group normalization, and axial attention in the decoder. It achieved an impressive Dice scores of 0.9275 for whole tumor (WT), 0.8781 for tumor core (TC), and 0.8451 for enhancing tumors (ET) [38]. The significant improvement in Dice scores from 2020 to 2021 can likely be attributed to the considerable expansion of the dataset that year, which provided a richer variety of training examples. In 2021, pioneering research introduced few-shot and zero-shot learning approaches to the BraTS dataset. For example, Zhang et al. proposed a self-supervised tumor segmentation method based on layer decomposition that achieved a Dice score of around 71.6% on BraTS2018 for whole tumor segmentation in a zero-shot setting—that is, without any task-specific fine-tuning. With limited annotated data (e.g., 10%, 20%, or 40% of the training set), the method consistently outperformed other self-supervised approaches, demonstrating robust performance in low-data regimes. When fully supervised fine-tuning is applied using 100% of the labeled data, the Dice score improves to about 86.1%. These comparisons underscored the potential of zero-shot and few-shot strategies to significantly reduce reliance on extensive manual annotations while maintaining strong segmentation performance [39].
**2022:** The winning ensemble combined DeepSeg, an improved nnU-Net, and DeepSCAN architectures, utilizing the STAPLE method for final predictions. Dice scores on the BraTS 2021 test dataset reached 0.9294 for whole tumors (WT), 0.8788 for tumor core (TC), and 0.8803 for enhancing tumors (ET). The model excelled on the Sub-Saharan Africa dataset, achieving Dice scores of 0.9737 for whole tumors, showcasing its adaptability to underrepresented populations. However, performance on the pediatric dataset highlighted the need for further specialization in this domain [40].
**2023:** In 2023, the BraTS challenge saw significant advancements in various tasks: For task 1: Adult Glioma Segmentation, an ensemble comprising nnU-Net, Swin UNETR, and the BraTS 2021 winning model excelled, utilizing advanced synthetic data augmentation techniques such as GANs and registration. This approach not only harnessed the strengths of CNNs and transformers but also achieved impressive Dice score results of 0.9005 for whole tumor (WT), 0.8673 for tumor core (TC), 0.8509 for enhancing tumor (ET), with an average of 0.8729 [41].
Notably a team topped several categories, securing first place in Meningioma, BraTS-Africa, and Brain Metastases, and achieving second in Adult Glioma and Pediatric Tumors. Their achievements in Meningioma included the highest DSC for ET at 0.899, TC at 0.904, and WT at 0.871, demonstrating the efficacy of their model across diverse tumor types and complexities. The success of their model was the use of the AutoSeg3D framework, a flexible, Pytorch-based platform optimized to scale across GPUs. It incorporated advanced training techniques, including five-fold training with models such as SegResNet, DiNTS, and SwinUNETR, with SegResNet being particularly pivotal, chosen for its accuracy and suitability for complex tasks [42].
The winner of the Pediatric Tumor Challenge employed a model ensemble technique. They addressed the task using an ensemble of nnU-Net and Swin UNETR, with a strategic focus on maximizing the effectiveness of segmentation across multiple tumor sub-regions. This method involved tailored ensemble strategies for different tumor regions, enhancing both the accuracy and robustness of the segmentation results [43].
The BraSyn Challenge for Synthesizing Missing MRI Sequences was addressed by two groups. The winning group explored multiple advanced loss functions to improve the quality and realism of synthesized MRI images. Their innovative approach, which combined multiple loss functions such as L1 norm, masked L1 loss, adversarial loss, and others, significantly enhanced the synthesis quality. This was evidenced by median Dice scores of 0.72 for ET, 0.78 for TC, and 0.44 for WT, with an overall mean SSIM of 0.817 for the test set [44].
The winning team of the Inpainting Challenge addressed the task using a 3D U-Net model optimized with MAE and SSIM loss functions, which yielded robust performance metrics: a median similarity index measure (SSIM) of 0.91, a median root mean square error (RMSE) of 0.07, and a median peak signal-to-noise ratio (PSNR) of 23.59. This task demonstrated the specialized yet critical role of inpainting in biomedical image analysis, highlighting the potential for significant advancements in diagnostic imaging techniques [29].
**2024:** In Task 1: Post-Treatment Glioma Segmentation, the same team that topped the rankings in Task 1 2023 continued their success by utilizing a sophisticated strategy with neural networks, specifically nnU-Net, MedNeXt, and Swin-UNETR. These networks were enhanced with synthetic data augmentation using GliGANs to boost tumor variability and balance class differences between healthy and diseased tissue. This strategy led to a broad improvement in the model’s generalization capabilities. The ensemble of models, including those trained on both real and synthetic data, produced balanced DSC validation results of 0.7557 for enhancing tumor (ET), 0.7868 for non-enhancing tumor core (NETC), 0.7053 for resection cavity (RC), 0.8704 for surrounding non-enhancing FLAIR hyperintensity (SNFH), 0.7500 for tumor core (TC), and 0.8734 for whole tumor (WT) [45].
The winning model for the BraTS Africa 2024 challenge used an ensemble approach combining nnU-Net and MedNeXt, adapted for the Sub-Saharan context with limited data quality and quantity. This involved a stratified fine-tuning strategy using both BraTS2023 Adult Glioma and BraTS-Africa datasets, focusing on radiomic analysis to refine the training process. The ensemble method and adaptive post-processing led to impressive validation test Dice scores of 0.870 for enhancing tumor (ET), 0.865 for tumor core (TC), and 0.926 for whole tumor (WT) [46].
The winning team of Task 7: Synthesis (Global)—missing MRI, used a novel two-stage model that significantly improved the synthesis of brain MRI images compared to 2023 results. Initially, the model synthesized MRI images from 2D slices using a new intensity encoding method, followed by a ‘Refiner’ stage using 3D volumetric data to enhance image quality. This approach not only mimicked original MRI sequences closely but also enhanced detail and accuracy in tumor depiction, achieving on a test set an SSIM of 0.8183, and Dice scores of 0.8399 for whole tumor (WT), 0.7133 for enhancing tumor (ET), and 0.7572 for tumor core (TC) [47].

The results from other BraTS 2024 challenges were not yet available or published.

To summarize the SOTA development over the years since 2014, the BraTS challenges have showcased significant advancements in brain tumor segmentation, primarily driven by the evolution of deep convolutional networks. This period marked the beginning of deep learning’s dominance in medical imaging, with U-Net and its derivatives becoming central to ongoing innovations. These models have continuously incorporated more sophisticated features such as residual connections, densely connected layers, and attention mechanisms, each enhancing the segmentation accuracy.

The adoption of nnU-Net in 2020 was a significant turning point for the BraTS competitions, setting a new standard with its robust framework tailored for medical imaging segmentation. nnU-Net incorporated features like enhanced data augmentation and batch Dice loss, significantly improving operational efficiency and segmentation accuracy.

By 2022 and 2023, the integration of transformer-based models with traditional CNNs began to reflect a trend toward hybrid approaches. These models aim to merge the spatial learning capabilities of CNNs with the contextual understanding provided by transformers, such as with the incorporation of Swin UNETR, to improve generalization and segmentation accuracy.

In 2024, the BraTS challenge leveraged established models such as nnU-Net, benchmarking them against newer architectures like MedNeXt. This approach highlights a continued effort to refine segmentation tools in response to evolving clinical scenarios, emphasizing the ongoing advancement in SOTA methodologies within the field. A prime example of such advancements is the recent development of BrainSegFounder, a 3D foundational framework for multimodal neuroimage segmentation. This model introduces a two-stage pretraining strategy using vision transformers, which significantly enhances its accuracy. Initially pretrained on a large-scale dataset featuring healthy brain scans, it undergoes further fine-tuning on the Brain Tumor Segmentation (BraTS) challenge datasets. This rigorous training regimen enables BrainSegFounder to demonstrate substantial performance gains, surpassing previous winning solutions that utilized fully supervised learning methods. Empirical comparisons show that BrainSegFounder outperforms established state-of-the-art models, including SwinUNETR, nn-UNET, SegResNet, TransBTS, and the brain segmentation foundation model published in Model-Zoo. The model’s superiority in these evaluations underscores the critical role of the BraTS datasets not only in benchmarking but also in enhancing the development of innovative, more effective segmentation technologies [48].

A comprehensive summary of these years’ advancements is provided in Table 4, which outlines the key changes in top-performing approaches from 2012 to 2024, including the evolution from traditional machine learning to advanced deep learning ensembles. Looking ahead to the future of brain tumor segmentation, the state of the art is expected to continue building on the strengths of established deep learning models while increasingly incorporating emerging technologies to boost adaptability and operational efficiency. Innovations such as federated learning, which has been gaining attention in recent BraTS iterations, could significantly transform the field by allowing models to learn from a vast network of decentralized data sources without compromising patient privacy. Additionally, advancements in explainable AI are poised to enhance the trustworthiness and interpretability of AI solutions in clinical settings. This progression ensures that these sophisticated tools not only support but also clarify the decision-making processes of medical professionals, making advanced diagnostics more accessible and understandable.

While the field has seen tremendous growth in terms of technical capabilities, the journey ahead involves further technological innovations, ensuring that these advancements are sustainable, equitable, and effectively integrated into diverse clinical workflows worldwide [41,45].

## 6. Datasets Growing Complexity over the Years

**Figure 14:**
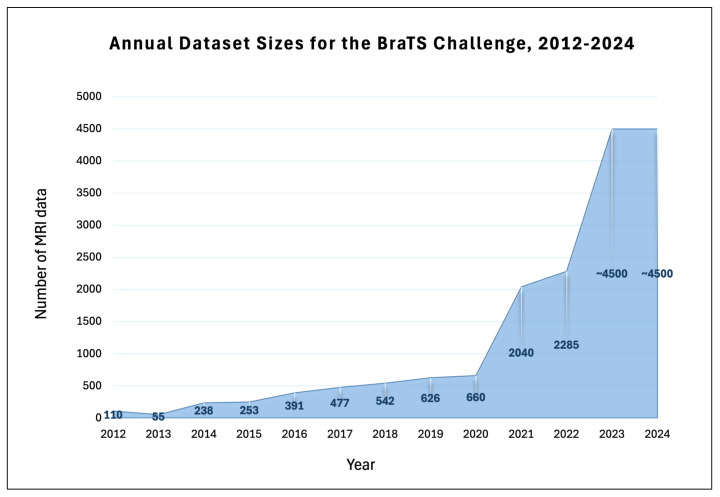
Annual expansion of the BraTS Challenge dataset sizes from 2012 to 2024, depicting the steady increase in the number of MRI scans utilized across training, validation, and test sets. The figure highlights critical growth phases, including the substantial expansion in 2021 due to increased institutional participation and dataset diversity, and the sharp rise in 2023, driven by the integration of new tumor subtypes and additional segmentation tasks. These trends reflect the growing complexity of the challenge and its increasing clinical relevance. The data for the graph were sourced from Table 1.

Since its inception in 2012, the BraTS challenge has undergone significant transformations in the complexity and scale of its datasets. This progression is visually represented in Figure 14, which illustrates the steady but exponential increase in dataset size from an initial 30 MRI scans in 2012 to approximately 4500 scans in 2024. The figure highlights key growth phases, with the years 2021 and 2023 showing the most significant increases. This growth exemplifies not only an increase in quantity but also significant enhancements in data variability and the precision of tumor annotations. The expansion in dataset size and the refinement in annotations stem from the increasing contributions from a diverse array of global institutions. This diversity is crucial as it introduces a wider range of imaging equipment and protocols, closely mirroring the real-world variability that robust medical imaging solutions must address.

Over the years, the challenge has progressively tackled more complex and clinically relevant problems. Recent datasets have expanded to include not only primary brain tumors but also intricate cases involving meningiomas and metastases. Furthermore, the inclusion of underrepresented populations, notably from Sub-Saharan Africa, adds a critical dimension to the challenge. It addresses global health disparities and enriches the representation in medical research, considering the unique glioma characteristics prevalent in these populations. Another significant challenge in these regions is the use of lower-quality MRI technology, which produces poorer image contrast and resolution, therefore making the annotation process more complex and demanding.

The inaugural 2012 BraTS dataset consisted of 30 high-resolution, multi-contrast MRI scans of glioma patients, accompanied by expert annotations for various tumor sub-regions. This dataset was supplemented by 50 synthetic images to provide additional training resources. Initially offering two class labels, it soon became evident that such categorization oversimplified the tumor’s complexity. The classification was thus expanded to include four subregions, necrosis, edema, non-enhancing, and enhancing tumor, setting a foundation for complex tumor segmentation. By 2013, the dataset structure was maintained but shifted towards greater clinical relevance by omitting synthetic cases. However, the limited number of datasets in 2012 and 2013 posed challenges like overfitting and limited clinical representativeness due to the lack of data diversity. The 2014 challenge significantly increased the dataset to about 300 new cases, reusing data from previous years to ensure continuity. Criticism arose due to biases introduced by using ground truth labels annotated by fusing results from top algorithms from earlier challenges. This led to the exclusion of these automatically labeled datasets in subsequent challenges, and by 2017, data from 2014 to 2016 were re-labeled by experts to ensure reliability. From 2014 to 2016, BraTS also introduced longitudinal datasets, adding a temporal dimension to the challenge. This required algorithms to not only segment tumors from static images but also to track changes across sequential scans.

A significant change occurred in 2017, when the number of class labels was reduced from four to three. This decision came after recognizing that the non-enhancing tumor (NET) was often overestimated due to unclear imaging evidence. Merging NET with the necrotic core (NCR) minimized subjective interpretations and led to more consistent and clinically relevant annotations across institutions. From 2017 onwards, the objective remained the same, but the dataset and number of contributing institutions grew annually. In 2020, the dataset included 660 multimodal pre-operative scans contributed by 19 institutions worldwide, emphasizing the need for algorithms that perform well across varied imaging standards.

By 2021, the adult glioma dataset had expanded substantially to 2040 cases, significantly enhancing the dataset’s demographic diversity. This growth was supported by over 40 annotators globally, increasing data diversity and representativeness. This year also introduced a new task of evaluating classification methods to predict MGMT promoter methylation status, aiding in precise glioblastoma diagnosis and treatment planning.

In 2023, the BraTS challenge reached a new zenith, with approximately 4500 cases from contributing institutions around the world, introducing nine tasks that span various populations and brain tumor types. Additionally, new segmentation labeling was introduced to better address the diverse tasks and clinical goals of these expanded datasets. In 2024, the challenge not only sustained its previous enhancements but also introduced post-treatment MRI data for the first time. Alongside this, a pathology task involving digitized histopathology images was added. These advancements significantly enhance the clinical relevance of the dataset, catering to both pre-treatment and post-treatment scenarios for a comprehensive approach to tumor management.

Each successive year has not only increased the volume of data but also refined the quality of annotations and the diversity of imaging scenarios. This evolution aims to enhance the algorithms’ generalizability from controlled datasets to real-world clinical scenarios, where variability in imaging protocols and tumor presentation could significantly influence automated segmentation outcomes.

## 7. Dataset Usage and Impact on Research: Potential Clinical Practice

**Figure 15:**
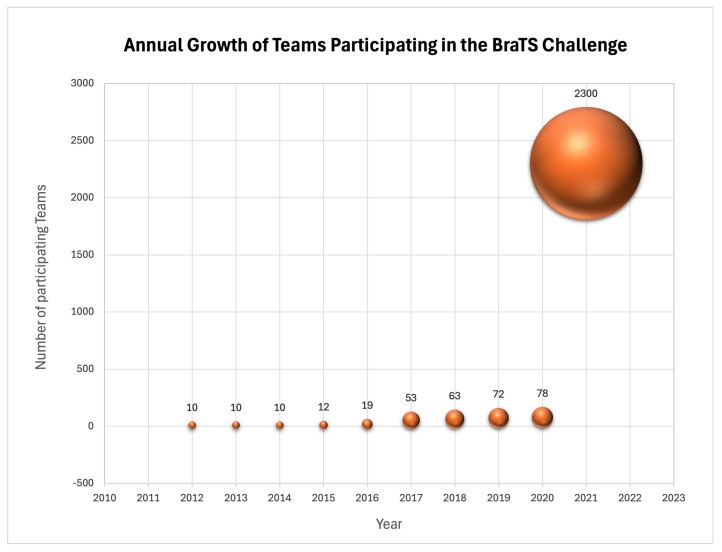
Graph illustrating the annual increase in the number of teams participating in the BraTS Challenge from 2012 to 2021. The figure highlights the exponential growth in participation, particularly in 2021, which saw an unprecedented jump to over 2300 teams−coinciding with the largest dataset expansion in BraTS history for a single task (adult glioma segmentation). The data employed are from [23].

The BraTS challenges have significantly advanced MRI-based glioma segmentation, potentially enhancing clinical diagnostics and treatment planning. These challenges continuously evolve, refining algorithms that precisely delineate tumor subregions—an essential endeavor for optimizing therapeutic strategies and monitoring disease progression. The multifaceted, high-quality, annotated multi-modal MRI datasets strive to reflect real-world complexities, though further improvements remain possible. They serve as invaluable tools for training and validating advanced segmentation models. The continuous increase in the number of participating teams in the BraTS challenges, which saw an exponential rise from 78 in 2020 to over 2300 in 2021, underscores the expanding global interest and importance of these datasets in advancing medical imaging technology and clinical practice. This surge closely coincided with a significant expansion of the dataset—more than tripling its size from the 2020 to the 2021 challenge—introducing the largest publicly available annotated dataset at that time. Figure 15 illustrates this sharp rise in participation, emphasizing how the substantial growth in dataset availability directly contributed to a surge in research interest. The dramatic increase in 2021 also reflects the growing recognition of BraTS as a benchmark for brain tumor segmentation, solidifying its role in the development of state-of-the-art medical AI applications. In the latest years, the addition of new tasks and scopes further boosted participation, as each edition continued to offer the largest publicly available datasets for its specific focus, thereby continually fostering deeper exploration and innovation in MRI-based brain tumor segmentation.

Over the years, BraTS has introduced a variety of tasks addressing different aspects of brain tumor treatment and patient care, such as longitudinal studies for tracking disease progression and predictive modeling for patient overall survival (OS) and MGMT promoter methylation status. The recent inclusion of underrepresented populations—specifically pediatric patients and Sub-Saharan African patients—along with a focus on diverse brain conditions such as meningiomas and metastases, broadens the global health applicability of these datasets. This enhancement in diversity is further validated by the fact that the datasets for pediatric patients and Sub-Saharan African patients represent the largest publicly available collections for these target groups [25,27]. Moreover, the Pathology challenge, initiated in 2024, enhances the ability to conduct detailed analyses of tumor heterogeneity through digitized tissue sections, thereby aiding in the development of more effective therapies. These specialized tasks are designed to integrate seamlessly into clinical workflows, equipping clinicians with a comprehensive toolkit for precise assessment of disease characteristics and progression. This ecosystem supports the development of patient-specific therapeutic strategies.

Particularly significant is the 2024 post-treatment glioma challenge, which emphasizes the important task of monitoring tumor changes after therapy, potentially fostering new research directions by revealing subtle morphological and molecular shifts post-treatment. This initiative aims to equip radiologists and neuro-oncologists with automated tools for consistent evaluation of tumor evolution, potentially streamlining the post-therapy monitoring process.

Furthermore, the challenges promote the development of robust segmentation models that perform consistently across various imaging protocols and institutional practices. This standardization is crucial for the incorporation of AI tools into clinical workflows, supporting real-time decision-making and enhancing the accuracy of brain tumor diagnostics and treatments. As BraTS editions continue to expand each year—to include larger patient cohorts and increasingly diverse pathologies—researchers will have broader opportunities to develop new segmentation architectures, compare evolving transfer learning strategies, and leverage molecular markers for a deeper understanding of tumor biology. These richer datasets can also inspire novel domain adaptation approaches that handle heterogeneous clinical environments, ultimately driving more generalized and reliable segmentation methods.

By aligning with precision medicine initiatives, these challenges enhance patient care and outcomes in neuro-oncology, showcasing the considerable clinical value of these complex and ever-growing datasets in a global healthcare context. Importantly, the inclusion of datasets from diverse regions—such as those collected in Sub-Saharan Africa with older, lower-quality imaging equipment—has driven the development of segmentation algorithms that are robust to variations in imaging protocols. In resource-limited settings, where advanced hardware may be scarce, such models can be deployed remotely: images acquired on legacy systems can be transmitted to centralized servers equipped with modern GPUs for processing, enabling high-quality diagnostics despite local limitations. This regional diversity in training data not only improves the generalizability of AI models but also fosters their adoption in varied healthcare environments, bridging gaps between well-resourced and under-resourced settings. This integrative approach, facilitated by the challenges, underscores the potential of precision medicine—developing tailored treatment plans based on comprehensive, individualized insights, thereby improving patient outcomes and optimizing healthcare delivery.

## 8. Challenges and Future Perspectives

Over the years, the Brain Tumor Segmentation (BraTS) challenges have revealed several persistent obstacles in developing datasets that can drive truly universal segmentation models. A recurring concern is the variability in MRI data quality, arising from institutions that use different scanners and clinical protocols, which leads to inconsistencies in image resolution, contrast, and acquisition parameters. Although more recent BraTS editions have introduced standardized pre-processing and annotation protocols to reduce intra-dataset variation, these very protocols can also limit the external applicability of trained models if the real-world data do not match the strict BraTS pipelines. Consequently, even the most extensive current dataset—such as the BraTS Adult Glioma Dataset, comprising over 1200 training images—does not guarantee robust performance when models trained on it are applied to clinical scenarios not represented within BraTS. In practice, such models often exhibit a performance drop compared to their BraTS test-set results, underscoring the impact of slight differences in pre-processing pipelines, image quality, or expert annotation guidelines outside the BraTS framework.

In our experiments, for instance, applying a state-of-the-art model called BrainSegFounder—extensively trained on BraTS Adult Glioma data—to an external dataset were provided, resulted in a Dice score of 0.755 on a skull-stripped version but only 0.094 on a non–skull-stripped version. This stark contrast highlights how seemingly minor variations (in this case, the presence of the skull in the input images) can substantially degrade performance. Fine-tuning the model on both versions of the external dataset improved performance, reaching a Dice score of 0.808 and 0.7886 for the skull-stripped and non–skull-stripped sets, respectively, yet these results still fall short of the 0.9069 attained on one of the BraTS Adult Glioma folders used for validation. Taken together, these outcomes demonstrate that while standardized protocols in BraTS are essential for consistency and comparability, they can introduce rigid assumptions that limit true generalizability across real-world data.

Recent expansions in BraTS, especially the inclusion of pediatric tumors and different brain conditions like meningiomas and brain metastases, have begun to address this gap. Yet, these additions also underscore the difficulty of capturing the unique characteristics and pathophysiology of such tumors, which often come with limited annotated data. Likewise, incorporating data from underrepresented regions (e.g., Sub-Saharan Africa) is pivotal for equitable healthcare but introduces complications considering lower-quality MRI technology, which yields images with inferior contrast and resolution, making it harder to annotate accurately.

Incomplete datasets further reflect real-world scenarios where not all four imaging modalities are available, emphasizing the need for advanced imputation or synthesis methods. The transition toward post-treatment segmentation, seen in the latest challenges, brings its own set of complexities due to radiation-induced changes, resected tumor beds, and other treatment artifacts—all of which demand more nuanced approaches to both annotation and algorithm design.

Looking ahead, future BraTS challenges could strengthen dataset generalization by broadening the standardized pre-processing pipeline to accommodate diverse imaging protocols, increasing the number and variety of contributing institutions, and promoting domain adaptation methods (i.e., techniques for handling data variations). They could also encourage AI-driven approaches—such as federated learning (training models across multiple institutions without sharing patient data) and transfer learning (adapting pretrained models to new conditions)—that are specifically designed to tackle heterogeneous clinical environments. Expanding longitudinal data to capture tumor evolution over multiple time points would support adaptive therapies and larger, more heterogeneous datasets—including rare tumor subtypes—can enhance the robustness of algorithms intended for broader clinical applications. Additionally, integrating multi-modal data sources (e.g., genetic, molecular, and radiomic information) would deepen insights into tumor biology, highlighting the need for global standardization in data acquisition and annotation. These approaches can alleviate privacy constraints, reduce bias, and foster models that are both generalizable and clinically relevant.

Finally, a continued commitment to inclusivity—encompassing diverse patient demographics, tumor types, and imaging quality—remains vital for bridging the gap between research innovations and real-world clinical benefits. Collaborative efforts within the global research community will be essential for refining annotation protocols, sharing resources, and establishing best practices, ensuring that future BraTS challenges not only push the boundaries of technical innovation but also pave the way for truly patient-centered, precision neuro-oncology solutions.

## 9. Conclusions

As demonstrated throughout this review, BraTS challenges from 2012 to 2024 have profoundly influenced the development of robust MRI-based glioma segmentation methods, growing from 30 scans to a rich, multi-institutional dataset exceeding 4500 cases. Below are the key findings and how they inform future research:

- BraTS Dataset Growth: Over a decade of challenges has seen a remarkable increase in dataset size, complexity, and diversity, fostering continual improvements in segmentation accuracy and clinical relevance. This growth is crucial for data-intensive deep learning methods.
- Standardized Pre-Processing Protocol: Introducing a uniform pre-processing pipeline enabled global data collection from institutions worldwide, ensuring diverse yet high-quality datasets. However, these standardized steps may also limit performance in real-world scenarios where protocols deviate from BraTS guidelines. Future research can leverage these pipelines to build larger, more consistent repositories while also exploring methods to maintain robustness under varying acquisition conditions.
- Standardized Annotation Protocol: Refined annotation methods and multi-rater consensus have enhanced the granularity and reliability of tumor segmentation. These consistent labels across different institutions reduce inter-observer variability and ensure more robust model training and evaluation.
- Inclusion and Representativeness: Recent expansions incorporate underrepresented populations (e.g., Sub-Saharan Africa, pediatric cases), new tumor types (meningiomas, metastases), and post-treatment datasets, supporting algorithmic generalization and advancing global health equity.
- Clinical Translation: Beyond boosting segmentation performance, these standardized datasets aim to streamline clinical workflows by saving clinician’s time and improving diagnostic and therapeutic planning. The datasets reinforce personalized treatment strategies, real-time monitoring, and post-treatment evaluation, aligning with the principles of precision medicine.
- Challenges and Limitations: Despite the substantial progress, obstacles like missing data, heterogeneous imaging protocols, and ensuring cross-institutional generalizability persist. Some BraTS subsets also remain relatively small and would benefit from further expansion.

This review fills a critical gap in the literature by providing the first comprehensive overview of the BraTS challenges from their inception to the most recent edition. In doing so, it tracks the evolution and improvements made over the years, offering detailed insights into all publicly available datasets released through these challenges. Researchers and practitioners alike can benefit from having a single, authoritative resource that compiles technical details, annotation protocols, and datasets, thereby streamlining access to crucial information on brain tumor segmentation. This in-depth perspective not only highlights key milestones in BraTS development but also serves as a springboard for future research, guiding further enhancements in dataset quality, annotation practices, and model performance in MRI-based neuro-oncology.

## Figures and Tables

**Table 1:** Summary of BraTS Challenge data distribution across training, validation, and test cohorts from 2012 to 2024, along with associated tasks and clinical timepoints. By tracking changes in dataset size, variety of tasks, and targeted clinical settings, this table underscores the growing complexity of BraTS challenges and illustrates how each year’s iteration provides a foundation for new research directions in MRI-based brain tumor segmentation. The data for the table were collected through extensive research of the challenges’ websites and related manuscripts.

Year	Total	Training	Validation	Testing	Tasks	Timepoint
	Data	Data	Data	Data		
2012	45 real	30	NA	15	Brain Tumor Segmentation	Pre-operative
	65 synthetic	50	NA	15		
2013	55	30	NA	25	Brain Tumor Segmentation	Pre-operative
2014	238	200	NA	38	Brain Tumor Segmentation	Longitudinal
					Disease Progression	
2015	253	200	NA	53	Brain Tumor Segmentation	Longitudinal
					Disease Progression	
2016	391	200	NA	191	Brain Tumor Segmentation	Longitudinal
					Disease Progression	
2017	477	285	46	146	Brain Tumor Segmentation	Pre-operative
					Overall Survival Prediction	
2018	542	285	66	191	Brain Tumor Segmentation	Pre-operative
					Overall Survival Prediction	
2019	626	335	125	166	Brain Tumor Segmentation	Pre-operarive
					Overall Survival Prediction	
					Quantification of Uncertanty	
2020	660	369	125	166	Brain Tumor Segmentation	Pre-operative
					Overall Survival Prediction	
					Quantification of Uncertanty	
2021	2040	1251	219	570	Brain Tumor Segmentation	Pre-operative
					MGMT classification	
2022	2285	1251	219	815	Brain Tumor Segmentation	Pre-operative
2023	2040	1251	219	570	Segmentation: Adult Glioma	Pre-operative
	105	60	15	30	Segmentation: BraTS-Africa	Pre-operative
	1424	1000	141	283	Segmentation: Meningioma	Pre-operative
	966	402	31	59	Segmentation: Brain Metastases	Pre-operative
	228	99	45	84	Segmentation: Pediatric Tumors	Pre-operative
	2040	1251	219	570	Synthesis (Global): Inpainting	Pre-operative
	2038	1251	219	568	Synthesis (Local): Inpainting	Pre-operative
	2040	1251	219	570	Evaluating Augmentations for BraTS	Pre-operative
2024	2200	1540	220	440	Segmentation: Adult Glioma Post Treatment	Post-operative
	/	60	35	/	Segmentation: BraTS-Africa	Pre-operative
	750	500	70	180	Segmentation: Meningioma Radiotherapy	Pre & Post-operative
	/	652	88	/	Segmentation: Brain Metastases	Pre & Post-operative
	464	261	91	112	Segmentation: Pediatric Tumors	Pre-operative
	/	2489	451	/	Segmentation: Generalizability	Pre-operative
	2099	1251	219	629	Synthesis (Global): Missing MRI	Pre-operative
	2315	1251	219	845	Synthesis (Local): Inpainting	Pre-operative
	2040	1251	219	570	Evaluating Augmentations for BraTS	Pre-operative
	280,000	195,000	25,000	60,000	Pathology	Pre-operative

**Table 2:** Summary of dataset composition across BraTS Challenges from 2014 to 2016. Although these datasets introduced longitudinal data and expanded upon earlier challenges, they were later discarded (from 2017 onward) and manually re-annotated by experts due to their reliance on algorithmic ground truth (GT) fusion. Table adapted and modified from [16].

Year	Total Data	Training Data	Validation Data	Test Data
**BraTS 2014**	238	200 data points: from BraTS 2012–2013 and TCIA, including longitudinal data	NA	38 unseen data points from BraTS 2012–2013 test set and TCIA
**BraTS 2015**	253	200 data points: identical to the BraTS 2014 training set	NA	53 unseen data points from BraTS 2012–2013 test set and TCIA
**BraTS 2016**	391	200 data points: identical to the BraTS 2014 training set	NA	191 unseen data points from BraTS 2012–2013 test set and TCIA

**Table 3:** Summary of BraTS Challenge details from 2012 to 2024, highlighting the primary tasks, MRI modalities included in each dataset, classes per task, the number of contributing institutions, and whether the dataset integrates patient data from low- and middle-income countries (LMICs). This overview underscores the evolving scope and global applicability of the BraTS challenges. The data for the table were collected through extensive research of the challenges’ websites and related manuscripts.

Year	Tasks	MRI	Classes	Contributing	LMICs
		Modalities	per Task	Institutions	
2012	Brain Tumor Segmentation	T1, T1c, T2, FLAIR	initial: 2	4	False
			final: 4		
2013	Brain Tumor Segmentation	T1, T1c, T2, FLAIR	4	4	False
2014	Brain Tumor Segmentation	T1, T1c, T2, FLAIR	4	5	False
	Disease Progression	/			
2015	Brain Tumor Segmentation	T1, T1c, T2, FLAIR	4	5	False
	Disease Progression		/		
2016	Brain Tumor Segmentation	T1, T1c, T2, FLAIR	4	5	False
	Disease Progression		/		
2017	Brain Tumor Segmentation	T1, T1c, T2, FLAIR	3	19	False
	Overall Survival Prediction	/			
2018	Brain Tumor Segmentation	T1, T1c, T2, FLAIR	3	19	False
	Overall Survival Prediction		/		
2019	Brain Tumor Segmentation	T1, T1c, T2, FLAIR	3	19	False
	Overall Survival Prediction		/		
	Quantification of Uncertanty		/		
2020	Brain Tumor Segmentation	T1, T1c, T2, FLAIR	3	19	False
	Overall Survival Prediction		/		
	Quantification of Uncertanty		/		
2021	Brain Tumor Segmentation	T1, T1c, T2, FLAIR	3	14	False
	MGMT classification		/		
2022	Brain Tumor Segmentation	T1, T1c, T2, FLAIR	3	14	False
2023	Segmentation: Adult Glioma	T1, T1c, T2, FLAIR	3	14	False
	Segmentation: BraTS-Africa	T1, T1c, T2, FLAIR	3	4	True
	Segmentation: Meningioma	T1, T1c, T2, FLAIR	3	6	False
	Segmentation: Brain Metastases	T1, T1c, T2, FLAIR	3	11	False
	Segmentation: Pediatric Tumors	T1, T1c, T2, FLAIR	3	8	False
	Synthesis (Global): Inpainting	T1, T1c, T2, FLAIR	/	14	False
	Synthesis (Local): Inpainting	T1	/	14	False
	Evaluating Augmentations for BraTS	T1, T1c, T2, FLAIR	3	14	False
2024	Segmentation: Adult Glioma Post Treatment	T1, T1c, T2, FLAIR	4	7	False
	Segmentation: BraTS-Africa	T1, T1c, T2, FLAIR	3	10	True
	Segmentation: Meningioma Radiotherapy	T1c	1	7	False
	Segmentation: Brain Metastases	T1, T1c, T2, FLAIR	3	10	False
	Segmentation: Pediatric Tumors	T1, T1c, T2, FLAIR	4	8	False
	Segmentation: Generalizability	T1, T1c, T2, FLAIR	3	/	False
	Synthesis (Global): Missing MRI	T1, T1c, T2, FLAIR	/	14	False
	Synthesis (Local): Inpainting	T1	/	14	False
	Evaluating Augmentations for BraTS	T1, T1c, T2, FLAIR	3	14	False
	Pathology	/	9	11	False

**Table 4:** Overview of the top-performing approaches in BraTS challenges from 2012 to 2024, highlighting the primary and emerging methods and architectures that achieved superior results each year. This chronological summary illustrates the evolution of segmentation techniques, from traditional machine learning approaches to state-of-the-art deep learning ensembles, emphasizing the continual advancement in accuracy and robustness of brain tumor segmentation.

Year	Top-Performing Approaches
**2012**	Predominance of Random Forests, logistic regression, and Markov Random Fields.
**2013**	Continuation of predominance of Random Forests-based methods.
**2014**	Deep convolutional network introduction alongside traditional RF methods. Emergence of U-Net, a CNN architecture.
**2015**	Domination of CNNs.
**2012**	Predominance of Random Forests, logistic regression, and Markov Random Fields.
**2016**	3D CNNs, DeepMedic, led the challenge.
**2017**	Predominance of U-Net and DeepMedic architectures. Ensemble methods became prominent.
**2018**	Dominant use of CNNs, particularly U-net architectures and their ensembles, which formed the basis of the top submissions.
**2019**	The Two-Stage Cascaded U-Net introduced a coarse-to-fine segmentation approach, winning the challenge.
**2020**	The nnU-Net framework dominated.
**2021**	The winning model extended nnU-Net, incorporating a larger network architecture, group normalization, and axial attention in the decoder.
**2022**	The winning ensemble combined DeepSeg, an improved nnU-Net, and DeepSCAN architectures, utilizing the STAPLE method for final predictions.
**2023**	Predominance of ensemble approaches combining CNNs (e.g., nnU-Net, SegResNet) with transformer architectures (e.g., Swin-UNETR). Widespread adoption of advanced data augmentation (GANs, registration, multi-fold training) and specialized tasks—like inpainting and MRI synthesis—employing innovative loss functions (e.g., adversarial, masked L1) to enhance realism and segmentation performance.
**2024**	Continued evolution through ensemble strategies and innovative synthetic augmentation. Ensembles that combine CNN-based models (e.g., nnU-Net, MedNeXt) with transformer architectures (e.g., Swin-UNETR) and enhanced by synthetic data augmentation using GliGANs emerged as top-performing submissions. For MRI synthesis, a novel two-stage model—first synthesizing MRI slices with an innovative intensity encoding and then refining with 3D volumetric data—achieved strong results.

## Data Availability

Not applicable.
